# Continuous flow synthesis of azobenzenes via Baeyer–Mills reaction

**DOI:** 10.3762/bjoc.18.78

**Published:** 2022-06-30

**Authors:** Jan H Griwatz, Anne Kunz, Hermann A Wegner

**Affiliations:** 1 Institute of Organic Chemistry, Justus Liebig University, Heinrich-Buff-Ring 17, 35392 Giessen, Germany; 2 Center for Material Research (ZfM/LaMa), Justus Liebig University, Heinrich-Buff-Ring 16, 35392 Giessen, Germany

**Keywords:** azobenzenes, Baeyer–Mills reaction, continuous flow, molecular switches, solar fuel

## Abstract

Azobenzene, as one of the most prominent molecular switches, is featured in many applications ranging from photopharmacology to information or energy storage. In order to easily and reproducibly synthesize non-symmetric substituted azobenzenes in an efficient way, especially on a large scale, the commonly used Baeyer–Mills coupling reaction was adopted to a continuous flow setup. The versatility was demonstrated with a scope of 20 substances and the scalability of this method exemplified by the synthesis of >70 g of an azobenzene derivative applied in molecular solar thermal storage (MOST) systems.

## Introduction

**Scheme 1:**
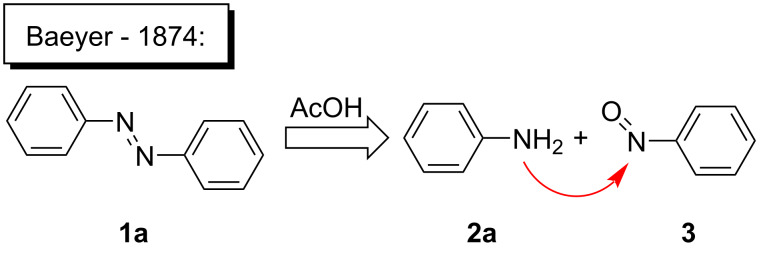
Baeyer–Mills reaction of AB (**1a**) involving nucleophilic attack of aniline (**2a**) to nitrosobenzene (**3**).

Although the red-colored azobenzenes (AB) have been known for years as dyes, their applications nowadays span from energy and information storage [1–5], organocatalysis [6], photobiology and photopharmacology [7], host–guest chemistry [8], molecular mechanics [9–10], to molecular machines [11]. This popularity is due to the ability of ABs to isomerize from their energetically more stable (*E*)- to the meta-stable (*Z*)-isomer by irradiation with light [12]. During this isomerization, not only the geometry is altered from the planar (*E*)-AB to its twisted (*Z*)-AB form, but also its properties change (e.g., dipole moment and polarity) [13–14]. Furthermore, the (*Z*)-AB can be reversibly switched back by visible light or thermally [15]. To synthesize ABs a variety of reactions can be chosen from, each having both advantages and disadvantages. The best synthesis must be individually selected for the respective use [16]. There are various ways to access symmetric and non-symmetric AB compounds in a convenient way in batch size, as it has been summarized in detail [16]. One example is the reliable synthesis of symmetric ABs in high yields via a Cu-catalyzed oxidative coupling of aniline derivatives [17]. This synthesis can be also used for the formation of non-symmetric AB, however, only for a selected set of anilines. One of the most applied methods to access non-symmetric azobenzenes is based on the condensation of nitrosobenzenes with anilines (Scheme 1). This so-called Baeyer–Mills reaction, which was first published by Baeyer in 1874 and further investigated by Mills, proceeds best for electron-rich anilines with electron-poor nitrosobenzenes. The reactivity can be rationalized by the proposed mechanism, which involves nucleophilic attack of the aniline on the nitrosobenzene derivatives in acidic or basic media (Scheme 1) [18–21]. However, in order to use azobenzenes as functional materials, access to a large-scale process is necessary. In this context continuous flow synthesis is frequently discussed as potential solution to address this challenge. This technique is neither limited by the size of the reaction vessel nor the stirring as the reagents are pumped continuously through the reactor. The set-up also allows precise control of the reaction time and temperature, which can lead to higher yields and purity [22]. Flow chemistry to prepare azobenzenes has been previously applied to the Cu-catalyzed synthesis of symmetric substituted AB derivatives [23–24]. However, non-symmetric substituted ABs are not accessible by this method in an efficient way. Herein, we report a continuous flow synthesis of non-symmetric AB compounds via the Baeyer–Mills reaction, which allows to obtain large quantities of products from different substrates in a fast and efficient manner.

## Results and Discussion

**Figure 1:**
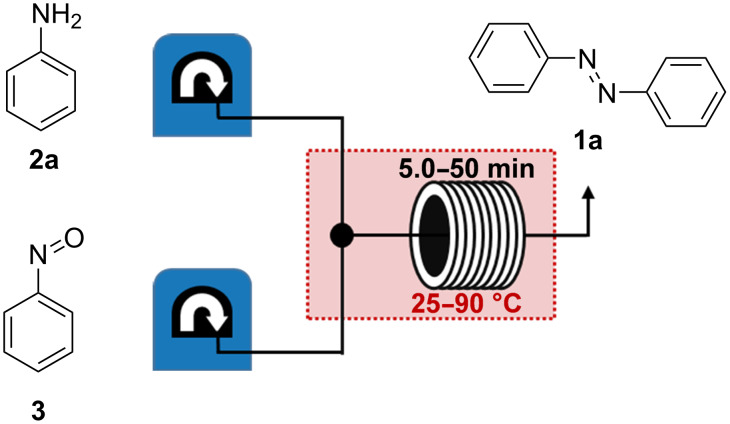
Flow setup for optimization of the Baeyer–Mills reaction with aniline (**2a**) and nitrosobenzene (**3**). In this setup the temperature of the tube reactor as well as the residence time can be individually varied (Figure 2).

For optimization of the Baeyer–Mills coupling in continuous flow the reaction to generate unsubstituted AB (**1a**) was performed with freshly distilled aniline (**2a**) and commercially available nitrosobenzene (**3**), dissolved separately in acetic acid. Both starting materials had the same concentration and were pumped by a Vapourtec E-Series system (for details, see experimental part in Supporting Information File 1). After mixing, the solution was passed through a tube reactor, in which the temperature as well as the residence time can be easily modified. Afterwards the respective reaction mixture was collected and analyzed (Figure 1). In order to optimize the reaction, both the temperature and the residence time were successively changed to achieve the highest conversion to AB. The residence time was increased from 5.0 to 50.0 min and the temperature was raised stepwise from 25 °C up to 90 °C (Figure 1). After the set residence time an aliquot was collected, diluted with acetonitrile, and subsequently examined by HPLC analysis (for details, see Supporting Information File 1).

**Figure 2:**
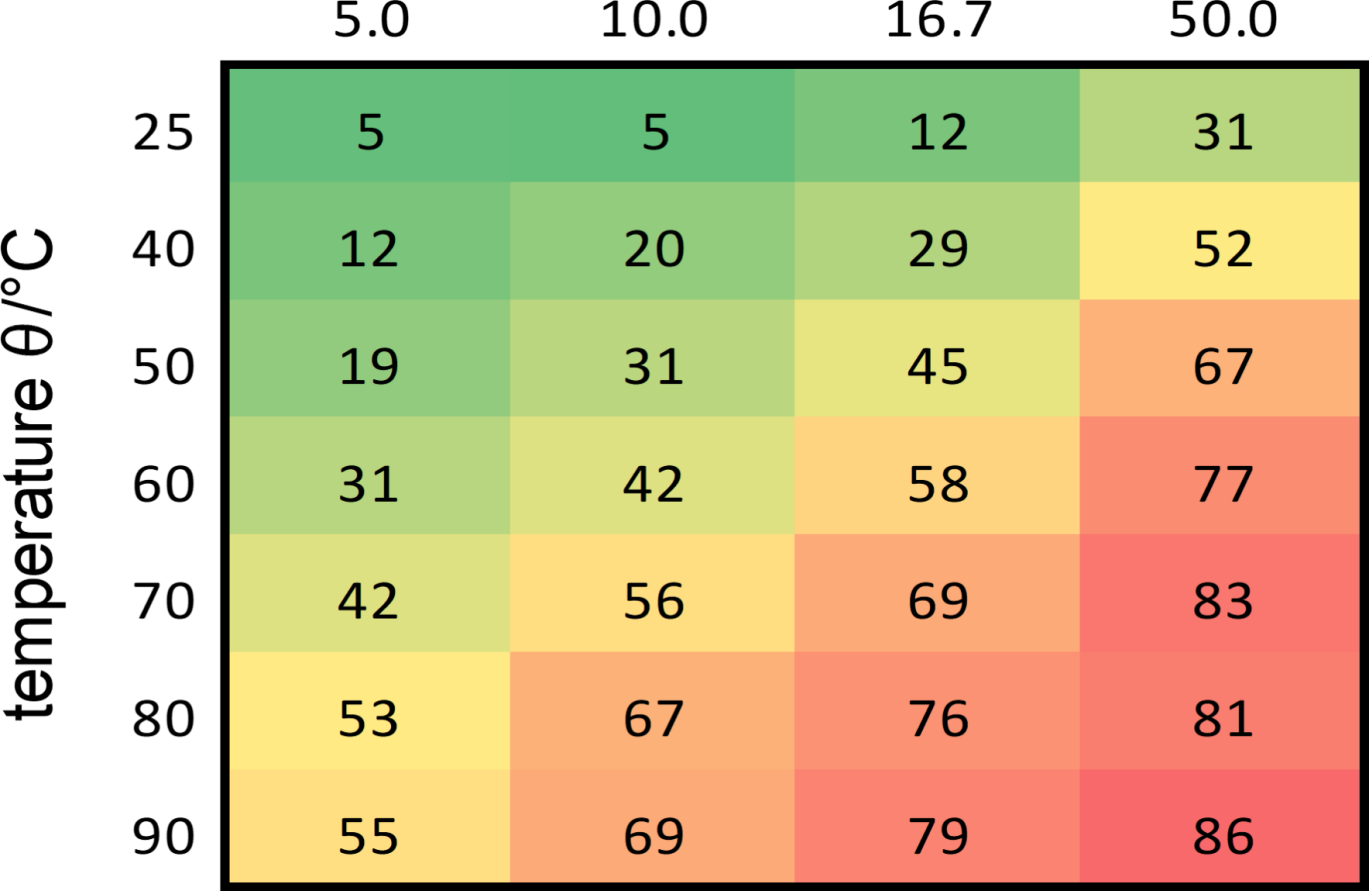
Optimization of the Baeyer–Mills reaction of nitrosobenzene (**3**) with aniline (**2a**) to AB (**1a**). The numbers in the figure result from the ratio of the integral of the HPLC peak of AB (**1a**) to the integral of AB (**1a**), nitrosobenzene (**3**), and aniline (**2a**) at a wavelength of 254 nm (see Supporting Information File 1 for details).

**Figure 3:**
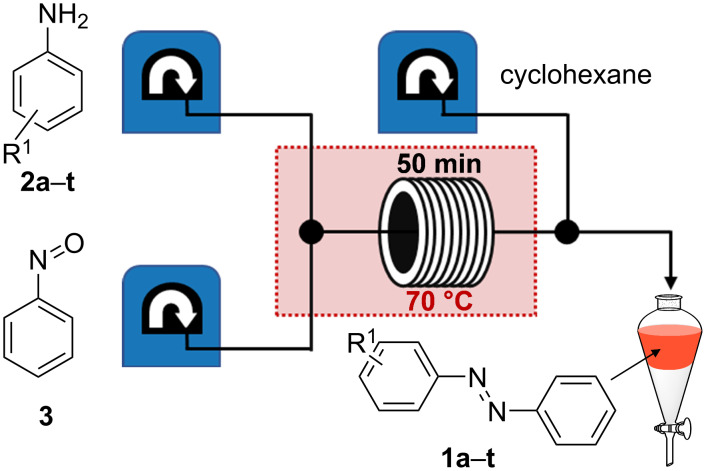
Flow setup after prior optimization with unsubstituted aniline (**2a**) and nitrosobenzene (**3**) to AB (**1a**).

At lower temperature, AB (**1a**) could be detected, but only a low conversion of the starting materials was observed (Figure 2). At higher temperature, the product/starting material ratio was improved but was still not satisfactory. Therefore, not only the temperatures, but also the residence time was gradually changed (Figure 2). At 70–90 °C and a residence time of 50.0 min the best results were observed. However, heating to 80–90 °C provided increasing amounts of azoxybenzene, which is a known side product of the Bayer–Mills reaction [25]. Hence, these parameters (70 °C, 50.0 min) were chosen to do the synthesis on a preparative scale. The setup was slightly modified to include an aqueous workup and extraction of the organic phase. For this purpose, a third pump was implemented which adds cyclohexane to the reaction mixture after the tube reactor (Figure 3). The reaction solution with cyclohexane was continuously fed into a separating funnel containing brine. After phase separation, drying of the organic phase with MgSO_4_, and evaporation of the solvent, AB (**1a**) could be obtained in 98% yield under the previously optimized conditions. By collection of the reactor output for 2 h, 582 mg of AB were obtained.

Further purification was not necessary as the product was satisfactorily pure with the described workup. After successful optimization of the synthesis of unsubstituted AB (**1a**) the setup was also tested for a large number of other azobenzene derivatives to determine the scope of the method (Table 1). All aniline derivatives **2a**–**s** were commercially available and the corresponding azobenzenes **1a**–**s** were synthesized according to the general procedure in continuous flow as described before. The optimized flow and workup conditions gave the products in high purity for most of the synthesized AB derivatives (see Supporting Information File 1 for details). Only in a few cases flash column chromatography was necessary to isolate the pure products (see Table 1). As expected, the method worked excellently for most of the electron-rich anilines due to their increased nucleophilicity.

**Table 1:** Substrate scope of the Baeyer–Mills reaction under the optimized conditions in continuous flow.

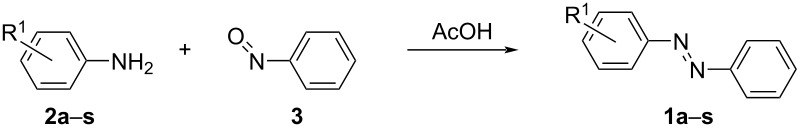			

entry	aniline **2**	product **1**	yield [%]

1	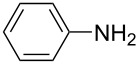 **2a**	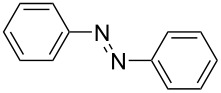 **1a**	98
2	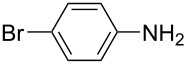 **2b**	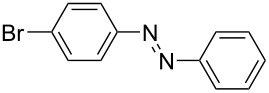 **1b**	89
3	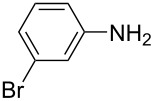 **2c**	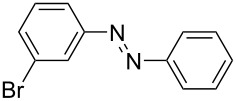 **1c**	77
4	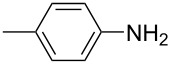 **2d**	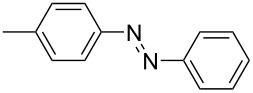 **1d**	94
5	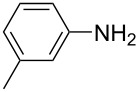 **2e**	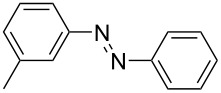 **1e** ^a^	79
6	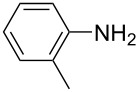 **2f**	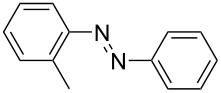 **1f** ^a^	67
7	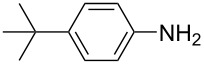 **2g**	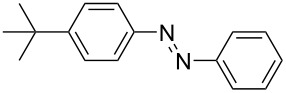 **1g**	>99
8	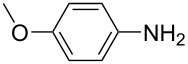 **2h**	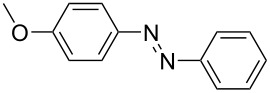 **1h**	96
9	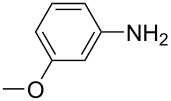 **2i**	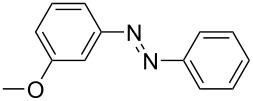 **1i** ^a^	7
10	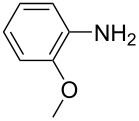 **2j**	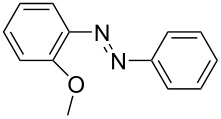 **1j** ^a^	72
11	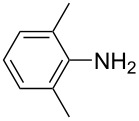 **2k**	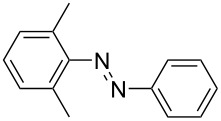 **1k** ^a^	23
12	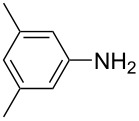 **2l**	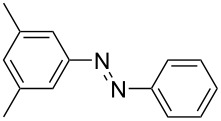 **1l** ^a^	65
13	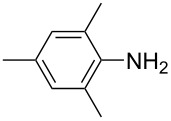 **2m**	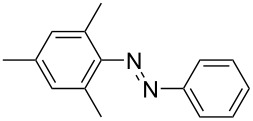 **1m**	70
14	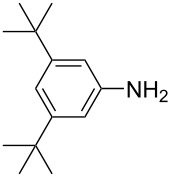 **2n**	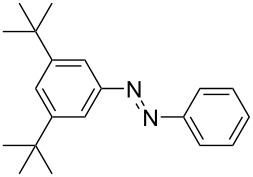 **1n**	99
15	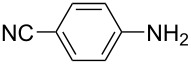 **2o**	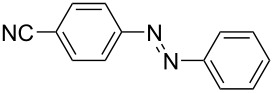 **1o**	7
16	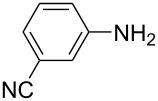 **2p**	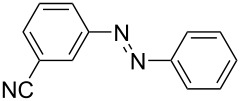 **1p**	54
17	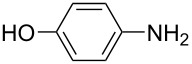 **2q**	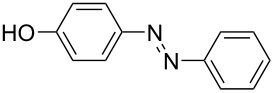 **1q**	68
18	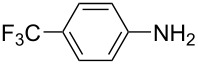 **2r**	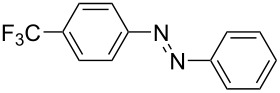 **1r** ^a^	33
19	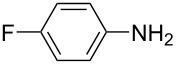 **1s**	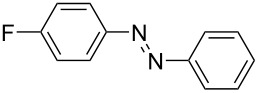 **1s**	95

^a^Purified by chromatography.

A comparison of *ortho-*, *meta*- and *para*-substituted derivatives revealed that for electron-rich anilines, the *para*-substituted ABs are formed in better yields as their *ortho-* and *meta*-analogues. For example, the synthesis of AB **1i** from *m*-anisidine (**2i**) gave only 7% yield, due to the formation of large amounts of azoxybenzene and purification issues therefrom. The moderate product yields from the *ortho*-substituted aniline derivatives are presumably caused by the higher steric hindrance of the nucleophilic attack. Low yields in case of electron-poor aniline derivatives can be explained by the reduced nucleophilicity. To exemplarily demonstrate the optimization for electron-poor derivatives, the synthesis of *p*-cyano-substituted AB **1o** was repeated at higher temperatures. Thereby the yield of **1o** could be increased to 17% (90 °C) and 19% (110 °C), respectively. However, since larger amounts of azoxybenzene were formed, column chromatography became necessary relativizing this improvement in yield (see Supporting Information File 1 for spectra). Substrates, which did not result in AB formation, were anilines with a nitro-substituent. Moreover, some further cases of *ortho*-substituted anilines were unsuccessful, for which steric effects could serve as an explanation (see Supporting Information File 1 for details). In comparison with published batch syntheses, the herein reported continuous flow synthesis usually gives similar or improved yields and eliminates the shortcomings in scalability.

**Scheme 2:**
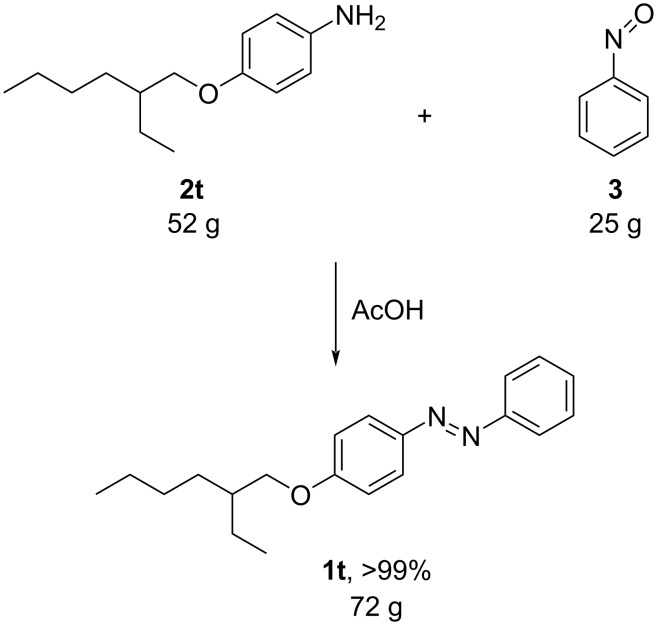
Large-scale synthesis of AB **1t** from aniline **2t** and nitrosobenzene (**3**) in >99% yield within 3 days.

For the applications of ABs as molecular materials often larger amounts are required, for example, as active compounds in molecular solar thermal energy storage (MOST) systems. Therefore, we utilized the set-up for the preparation of large amounts of AB **1t** (Scheme 2). This AB analogue was first synthesized by Masutani et al. in 2014 and was examined by them as well as in further studies by other groups regarding their potential for MOST applications, e.g., in a fluidic chip device by Wang et al. [1,26–27]. For the large-scale synthesis both, aniline **2t** as well as nitrosobenzene (**3**), were dissolved in acetic acid and, as described before, pumped through the flow setup (Figure 3). Every 12 h, the organic phase was separated from the aqueous phase, dried over MgSO_4_, and the solvent was subsequently removed. The solvent was recycled to minimize waste. After a total runtime of 3 days 72 g of AB **1t** were obtained as pure red oil which corresponds to a yield of >99%. Therefore, the method should be suitable for the preparation of easily several 100 grams of azobenzene compounds.

## Conclusion

In summary, the Baeyer–Mills reaction was successfully transferred to a continuous flow setup. The method can be used for various anilines as starting materials to access the desired ABs. A scope of 20 different anilines (**2a**–**t**) resulting in the corresponding azobenzenes **1a**–**t** was investigated and especially electron-rich azobenzenes were prepared in yields up to >99%. Furthermore, the setup was demonstrated to be applicable for a large-scale synthesis, where azobenzene **1t** was obtained in 72 g within 3 days without the need of further purification. With this process a large number of non-symmetric substituted azobenzenes can be prepared in high yields and large quantities which opens new possibilities for applications of AB as molecular materials in general.

## Supporting Information

File 1 General information, experimental data of all isolated products, ^1^H and ^13^C NMR spectra, and structures of unsuccessful substrates.
