# Supplementary material for: Continuous flow synthesis of azobenzenes via Baeyer–Mills reaction
Source: Beilstein J Org Chem. 2022 Jun 30;18:781–7. doi: 10.3762/bjoc.18.78 (PMC9263553; doi:10.3762/bjoc.18.78)
Supplement: File 1 — General information, experimental data of all isolated products, 1H and 13C NMR spectra, and structures of unsuccessful substrates. [file Beilstein_J_Org_Chem-18-781-s001.pdf]

BEILSTEIN JOURNAL OF ORGANIC CHEMISTRY

Supporting Information

for

Continuous flow synthesis of azobenzenes via Baeyer–Mills reaction

Jan H. Griwatz, Anne Kunz and Hermann A. Wegner

Beilstein J. Org. Chem. **2022,** *18,* 781–787. doi:10.3762/bjoc.18.78

General information, experimental data of all isolated products, ^{1}H and ^{13}C NMR spectra, and structures of unsuccessful substrates

License and Terms: This is a supporting information file under the terms of the Creative Commons Attribution License (https://creativecommons.org/licenses/by/4.0). Please note that the reuse, redistribution and reproduction in particular requires that the author(s) and source are credited and that individual graphics may be subject to special legal provisions.
The license is subject to the *Beilstein Journal of Organic Chemistry* terms and conditions: (https://www.beilstein-journals.org/bjoc/terms)

Table of contents

General information S3
Synthetic procedures S4
Optimization of reaction conditions S4
Synthesis of AB 1a S4
General procedure for the synthesis of AB derivatives **1b–s** S5
1-(4-Bromophenyl)-2-phenyldiazene (**1b**) S5
1-(3-Bromophenyl)-2-phenyldiazene (**1c**) S5
1-(4-tolyl)-2-phenyldiazene (**1d**) S5
1-(*3*-tolyl)-2-phenyldiazene (**1e**) S6
1-Phenyl-2-(*o*-tolyl)diazene (**1f**) S6
1-(4-(*tert*-Butyl)phenyl)-2-phenyldiazene (**1g**) S6
1-(4-Methoxyphenyl)-2-phenyldiazene (**1h**) S6
1-(3-Methoxyphenyl)-2-phenyldiazene (**1i**) S7
1-(2-Methoxyphenyl)-2-phenyldiazene (**1j**) S7
1-(2,6-Dimethylphenyl)-2-phenyldiazene (**1k**) S7
1-(3,5-Dimethylphenyl)-2-phenyldiazene (**1l**) S7
1-Mesityl-2-phenyldiazene (**1m**) S8
1-(3,5-Di-*tert*-butylphenyl)-2-phenyldiazene (**1n**) S8
4-(2-Phenyldiazenyl)benzonitrile (**1o**) S8
3-(2-Phenyldiazenyl)benzonitrile (**1p**) S8
4-Phenylazophenol (**1q**) S9
1-Phenyl-2-(4-(trifluoromethyl)phenyl)diazene (**1r**) S9
1-(4-Fluorophenyl)-2-phenyldiazene (**1s**) S9
Large scale synthesis of **1t** S10
Unsuccessful substrates (did not result in AB formation) S11
NMR spectra S12
Azobenzene (**1a**) S13
1-(4-Bromophenyl)-2-phenyldiazene (**1b**) S14
1-(3-Bromophenyl)-2-phenyldiazene (**1c**) S15
1-(4-Tolyl)-2-phenyldiazene (**1d**) S16
1-(3-Tolyl)-2-phenyldiazene (**1e**) S17
1-Phenyl-2-(*o*-tolyl)diazene (**1f**) S18
1-(4-(*tert*-Butyl)phenyl)-2-phenyldiazene (**1g**) S19
1-(4-Methoxyphenyl)-2-phenyldiazene (**1h**) S20
1-(3-Methoxyphenyl)-2-phenyldiazene (**1i**) S21
1-(2-Methoxyphenyl)-2-phenyldiazene (**1j**) S22
1-(2,6-Dimethylphenyl)-2-phenyldiazene (**1k**) S23
1-(3,5-Dimethylphenyl)-2-phenyldiazene (**1l**) S24
1-Mesityl-2-phenyldiazene (**1m**) S25

1-(3,5-Di-*tert*-butylphenyl)-2-phenyldiazene (**1n**) S26
4-(2-Phenyldiazenyl)benzonitrile (**1o**) S27
3-(2-Phenyldiazenyl)benzonitrile (**1p**) S28
4-Phenylazophenol (**1q**) S29
1-Phenyl-2-(4-(trifluoromethyl)phenyl)diazene (**1r**) S30
1-(4-Fluorophenyl)-2-phenyldiazene (**1s**) S31
1-[4-[(2-Ethylhexyl)oxy]phenyl]-2-phenyldiazene (**1t**) S32
4-(2-Phenyldiazenyl)benzonitrile (**1o**) – temperature screening S33
References S33

General information

***Chemicals**:* The chemicals were purchased from Sigma-Aldrich, Acros Organics, Alfa Aesar, and TCI Europe. Deuterated solvents were purchased from Euriso-Top GmbH. Technical grade solvents, used during work-up and purification, were distilled prior to use. Aniline **2t** was prepared according to the literature.[1]

***NMR spectroscopy**:* NMR spectra were measured on a Bruker Avance II 200 MHz, Avance II 400 MHz, Avance III 400 MHz HD, or Avance III 600 MHz spectrometer at 25 °C if not otherwise noted. As the internal reference, the shifts of the residual solvent peaks were used. For all azobenzenes, the thermodynamically more stable (*E*)-isomer is reported if not noted otherwise. Additional small peaks can be attributed to small amounts of (*Z*)-isomers. Chemical shifts are reported in parts per million (ppm) relative to the solvent peak, coupling constants (*J*) are reported in hertz (Hz).

Flow setup: The reactions were performed using a Vapourtec *E-Series* System (www.vapourtec.com). The reactions were performed in a tubular reactor with an inner volume of 10 mL, if not noted otherwise. FEP-tubing (inner diameter 0.75 mm, purchased from Techlab) was used for all other connections.

HPLC: HPLC analysis was performed using a Shimadzu LCMS-2020 system equipped with a 150 × 4 mm Eurospher 100-5 C18 column with acetonitrile/water 90:10 as isocratic eluent (1.5 mL/min). Detection was carried out using a Shimadzu SPD-M20A diode array detector at 254 nm.

Chromatography: Flash column chromatography was carried out with Silica 60 M (0.04–0.063 mm) from Macherey Nagel GmbH & Co. KG. Thin layer chromatography was performed on Polygram® SIL G/UV_{254} from Macherey Nagel GmbH & Co. KG.

Synthetic procedures

Optimization of reaction conditions

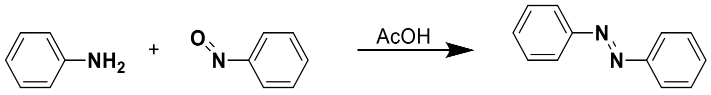

2a 3 1a

Aniline (**2a**, 1.50 g, 13.6 mmol, 97%, 1.00 equiv) and nitrosobenzene (**3**, 1.26 g, 13.6 mmol, 1.00 equiv) were each dissolved in acetic acid (99%, 50 mL respectively) in separate bottles and degassed by a nitrogen stream for 15 min. The flow rates for both reagents were screened (ratio always 1:1) for 1.00 mL/min (5 min residence time), 500 µL/min (10 min residence time), 300 µL/min (16.67 min residence time), 100 µL/min (50 min residence time). For each flow rate different temperatures (25 °C, 40 °C, 50 °C, 60 °C, 70 °C, 80 °C, and 90 °C) were screened. After the calculated residence time and a short equilibration time, an aliquot of ca. 1 mL was collected and after dilution with acetonitrile, HPLC analysis was performed. To have a relative quantification, the integral of the azobenzene was divided by the sum of all starting material and product peak integrals at a detector wavelength of 254 nm.

$$x = \frac{\text{peak area}_{254\ \text{nm}}\ (\mathbf{1a})}{\text{total peak area}_{254\ \text{nm}}}$$

Here, it is to be mentioned that we are aware that this procedure does not provide an absolute quantification of conversion, but it was a convenient and fast method, which could give a general trend as well as good estimation about the best reaction conditions by a relative quantification.

Synthesis of AB 1a

Aniline (**2a**, 1.50 g, 13.6 mmol, 97%, 1.00 equiv) and nitrosobenzene (**3**, 1.26 g, 13.6 mmol, 1.00 equiv) were each dissolved in acetic acid (99%, 50 mL respectively) in separate bottles and degassed by a nitrogen stream for 15 min. Additionally, a third bottle with cyclohexane was prepared. Both starting material solutions were pumped individually at 0.1 mL/min. The streams were mixed in a T-mixer and fed into a tubular reactor (10 mL) at 70 °C. After passing the reactor, the reaction mixture was diluted by cyclohexane (1.0 mL/min). After the calculated residence time and a short equilibration time, the output was collected for 120 min. The reaction mixture was fed into brine (100 mL) in a separatory funnel (see Figure S1). The organic layer was separated, dried with $MgSO_4$, and the solvent was removed under reduced pressure. AB **1a** was obtained as orange solid in a yield of 98% (582 mg, 3.19 mmol, contains <2% azoxybenzene).

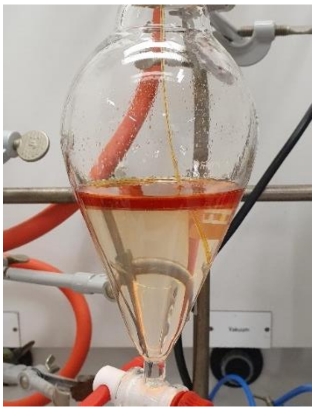

Figure S1: Collection of reactor output in a separatory funnel filled with brine.

Analytical data corresponds to literature.[2]

^{1}H NMR (600 MHz, $CDCl_3$) δ 7.95 – 7.91 (m, 4H), 7.55 – 7.51 (m, 4H), 7.50 – 7.46 (m, 2H).

^{13}C{^{1}H} NMR (151 MHz, $CDCl_3$) δ 152.8, 131.1, 129.2, 123.0.

General procedure for the synthesis of AB derivatives 1b–s

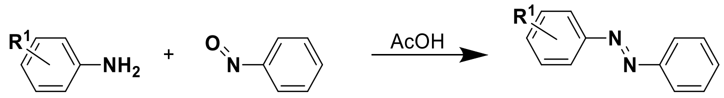

Aniline (**2b**–**s**, 1.00 equiv) and nitrosobenzene (**3**, 1.00 equiv) were each dissolved in acetic acid (99%, 10 mL respectively, if not stated otherwise) in separate bottles and degassed by a nitrogen stream for 15 min. Additionally, a third bottle with cyclohexane was prepared. Both starting material solutions were pumped individually at 0.1 mL/min. The streams were mixed in a T-piece and fed into a tubular reactor (10 mL) at 70 °C. After passing the reactor, the reaction mixture was diluted by cyclohexane (1.0 mL/min). After the calculated residence time and a short equilibration time, the output was collected for a given time. The reaction mixture was fed into brine (100 mL) in a separatory funnel (see Figure S1). The organic layer was separated, dried with $MgSO_4$, and the solvent was removed under reduced pressure.

1-(4-Bromophenyl)-2-phenyldiazene (1b)
p-Bromoaniline (**2b**, 1.21 g, 6.79 mmol, 97%) was dissolved in acetic acid (25 mL). Nitrosobenzene (**3**, 750 mg, 6.79 mmol, 97%) was dissolved in acetic acid (25 mL). The reaction was performed following the general procedure and the output was collected for 120 min. AB **1b** was obtained as an orange solid in a yield of 89% (753 mg, 2.88 mmol, contains <3% azoxybenzene).

Analytical data corresponds to literature.[3]

^{1}H NMR (600 MHz, $CDCl_3$)	δ 7.93 – 7.89 (m, 2H), 7.83 – 7.78 (m, 2H), 7.68 – 7.62 (m, 2H), 7.55 – 7.47 (m, 3H).
$^{13}C\{^{1}H\}$ NMR (151 MHz, $CDCl_3$)	δ 152.5, 151.5, 132.5, 131.5, 129.2, 125.4, 124.4, 123.0.

1-(3-Bromophenyl)-2-phenyldiazene (1c)
m-Bromoaniline (**2c**, 1.19 g, 6.79 mmol, 98%) was dissolved in acetic acid (25 mL). Nitrosobenzene (**3**, 750 mg, 6.79 mmol, 97%) was dissolved in acetic acid (25 mL). The reaction was performed following the general procedure and the output was collected for 120 min. AB **1c** was obtained as a red solid in a yield of 77% (655 mg, 2.51 mmol, contains <4% azoxybenzene).

Analytical data corresponds to literature.[4]

^{1}H NMR (600 MHz, $CDCl_3$)	δ 8.06 (t, *J* = 1.9 Hz, 1H), 7.94 – 7.90 (m, 2H), 7.88 (ddt, *J* = 7.9, 1.6, 0.7 Hz, 1H), 7.60 (ddt, *J* = 8.0, 1.8, 0.8 Hz, 1H), 7.56 – 7.48 (m, 3H), 7.40 (t, *J* = 7.9 Hz, 1H).
$^{13}C\{^{1}H\}$ NMR (151 MHz, $CDCl_3$)	δ 153.7, 152.5, 133.7, 131.7, 130.6, 129.3, 124.8, 123.3, 123.2, 123.1.

1-(4-tolyl)-2-phenyldiazene (1d)
p-Toluidine (**2d**, 294 mg, 2.72 mmol, 99%) was dissolved in acetic acid (10 mL). Nitrosobenzene (**3**, 300 mg, 2.72 mmol, 97%) was dissolved in acetic acid (10 mL). The reaction was performed following the general procedure and the output was collected for 60 min. AB **1d** was obtained as an orange solid in a yield of 94% (300 mg, 1.53 mmol, contains <2% azoxybenzene).

Analytical data corresponds to literature.[4]

^{1}H NMR (400 MHz, $CDCl_3$)	δ 7.92 – 7.87 (m, 2H), 7.86 – 7.81 (m, 2H), 7.54 – 7.43 (m, 3H), 7.35 – 7.29 (m, 2H), 2.44 (s, 3H).
$^{13}C\{^{1}H\}$ NMR (101 MHz, $CDCl_3$)	δ 152.9, 150.9, 141.7, 130.8, 129.9, 129.2, 123.0, 122.9, 21.7.

1-(*3*-tolyl)-2-phenyldiazene (1e)
m-Toluidine (**2e**, 291 mg, 2.72 mmol) was dissolved in acetic acid (10 mL). Nitrosobenzene (**3**, 300 mg, 2.72 mmol, 97%) was dissolved in acetic acid (10 mL). The reaction was performed following the general procedure and the output was collected for 60 min. The crude product was purified by flash column chromatography (SiO_2, cyclohexane/toluene, 1:1). AB **1e** was obtained as a red oil in a yield of 79% (254 mg, 1.29 mmol, contains <1% azoxybenzene).

Analytical data corresponds to literature.[4]

^{1}H NMR (400 MHz, $CDCl_3$) δ 7.96 – 7.86 (m, 2H), 7.75 – 7.71 (m, 2H), 7.55 – 7.43 (m, 3H), 7.43 – 7.37 (m, 1H), 7.31 – 7.26 (m, 1H), 2.46 (s, 3H).

^{13}C{^{1}H} NMR (101 MHz, $CDCl_3$) δ 152.9, 152.9, 139.1, 131.9, 131.0, 129.2, 129.0, 123.1, 122.9, 120.6, 21.5.

1-Phenyl-2-(*o*-tolyl)diazene (1f)
o-Toluidine (**2f**, 291 mg, 2.72 mmol) was dissolved in acetic acid (10 mL). Nitrosobenzene (**3**, 300 mg, 2.72 mmol, 97%) was dissolved in acetic acid (10 mL). The reaction was performed following the general procedure and the output was collected for 60 min. The crude product was purified by flash column chromatography (SiO_2, cyclohexane/toluene, 1:1). AB **1f** was obtained as an orange solid in a yield of 67% (216 mg, 1.10 mmol, contains <1% azoxybenzene).

Analytical data corresponds to literature.[4]

^{1}H NMR (400 MHz, $CDCl_3$) δ 7.97 – 7.92 (m, 2H), 7.68 – 7.62 (m, 1H), 7.57 – 7.46 (m, 3H), 7.39 – 7.33 (m, 2H), 7.32 – 7.24 (m, 1H), 2.75 (s, 3H).

^{13}C{^{1}H} NMR (101 MHz, $CDCl_3$) δ 153.1, 150.9, 138.3, 131.4, 131.1, 130.9, 129.2, 126.6, 123.1, 115.6, 17.7.

1-(4-(*tert*-Butyl)phenyl)-2-phenyldiazene (1g)
4-*tert*-Butylaniline (**2g**, 414 mg, 2.72 mmol, 98%) was dissolved in acetic acid (10 mL). Nitrosobenzene (**3**, 300 mg, 2.72 mmol, 97%) was dissolved in acetic acid (10 mL). The reaction was performed following the general procedure and the output was collected for 60 min. AB **1g** was obtained as a red solid in a yield of >99% (389 mg, 1.63 mmol, contains <1% azoxybenzene).

Analytical data corresponds to literature.[4]

^{1}H NMR (400 MHz, $CDCl_3$) δ 7.95 – 7.83 (m, 4H), 7.59 – 7.42 (m, 5H), 1.39 (s, 9H).

^{13}C{^{1}H} NMR (101 MHz, $CDCl_3$) δ 154.6, 152.9, 150.7, 130.7, 129.1, 126.0, 122.8, 122.6, 35.0, 31.3.

1-(4-Methoxyphenyl)-2-phenyldiazene (1h)
p-Anisidine (**2h**, 338 mg, 2.72 mmol, 99%) was dissolved in acetic acid (10 mL). Nitrosobenzene (**3**, 300 mg, 2.72 mmol, 97%) was dissolved in acetic acid (10 mL). The reaction was performed following the general procedure and the output was collected for 60 min. AB **1h** was obtained as a red solid in a yield of 96% (332 mg, 1.56 mmol, contains <2% azoxybenzene).

Analytical data corresponds to literature.[4]

^{1}H NMR (400 MHz, $CDCl_3$) δ 7.96 – 7.91 (m, 2H), 7.90 – 7.86 (m, 2H), 7.54 – 7.47 (m, 2H), 7.47 – 7.41 (m, 1H), 7.05 – 6.99 (m, 2H), 3.90 (s, 3H).

^{13}C{^{1}H} NMR (101 MHz, $CDCl_3$) δ 162.2, 152.9, 147.2, 130.5, 129.2, 124.9, 122.7, 114.4, 55.7.

1-(3-Methoxyphenyl)-2-phenyldiazene (1i)
m-Anisidine (**2i**, 1.19 g, 2.72 mmol, 98%) was dissolved in acetic acid (10 mL). Nitrosobenzene (**3**, 300 mg, 2.72 mmol, 97%) was dissolved in acetic acid (10 mL). The reaction was performed following the general procedure and the output was collected for 60 min. The crude product was purified by flash column chromatography (SiO_2, cyclohexane/ethyl acetate, 0–10%). AB **1i** was obtained as a red oil in a yield of 7% (25 mg, 2.5 mmol, contains <3% azoxybenzene).

Analytical data corresponds to literature.[5]

^{1}H NMR (400 MHz, $CDCl_3$)	δ 7.96 – 7.91 (m, 2H), 7.58 (ddd, *J* = 7.8, 1.8, 1.0 Hz, 1H), 7.56 – 7.41 (m, 5H), 7.06 (ddd, *J* = 8.2, 2.6, 1.0 Hz, 1H), 3.91 (s, 3H).
^{13}C{^{1}H} NMR (101 MHz, $CDCl_3$)	δ 160.5, 154.0, 152.7, 131.2, 129.9, 129.2, 123.0, 117.9, 117.3, 105.9, 55.6.

1-(2-Methoxyphenyl)-2-phenyldiazene (1j)
o-Anisidine (**2j**, 342 mg, 2.72 mmol, 98%) was dissolved in acetic acid (10 mL). Nitrosobenzene (**3**, 300 mg, 2.72 mmol, 97%) was dissolved in acetic acid (10 mL). The reaction was performed following the general procedure and the output was collected for 60 min. The crude product was purified by flash column chromatography (SiO_2, cyclohexane/toluene, 1:1). AB **1j** was obtained as a red oil in a yield of 72% (250 mg, 1.18 mmol, contains <1% azoxybenzene).

Analytical data corresponds to literature.[6]

^{1}H NMR (400 MHz, $CDCl_3$)	δ 7.96 – 7.88 (m, 2H), 7.67 (dd, *J* = 8.0, 1.7 Hz, 1H), 7.58 – 7.36 (m, 5H), 7.10 (dd, *J* = 8.4, 1.2 Hz, 1H), 7.03 (ddd, *J* = 8.3, 7.3, 1.2 Hz, 1H), 4.03 (s, 3H).
^{13}C{^{1}H} NMR (101 MHz, $CDCl_3$)	δ 157.1, 153.3, 142.4, 132.6, 130.9, 129.2, 123.1, 120.9, 117.1, 112.9, 56.5.

1-(2,6-Dimethylphenyl)-2-phenyldiazene (1k)
2,6-Dimethylaniline (**2k**, 333 mg, 2.72 mmol, 99%) was dissolved in acetic acid (10 mL). Nitrosobenzene (**3**, 300 mg, 2.72 mmol, 97%) was dissolved in acetic acid (10 mL). The reaction was performed following the general procedure and the output was collected for 60 min. The crude product was purified by flash column chromatography (SiO_2, cyclohexane/ethyl acetate, 0–10%). AB **1k** was obtained as a red oil in a yield of 23% (78 mg, 0.37 mmol, contains <1% azoxybenzene).

Analytical data corresponds to literature.[7]

^{1}H NMR (400 MHz, $CDCl_3$)	δ 7.93 – 7.88 (m, 2H), 7.57 – 7.49 (m, 4H), 7.17 – 7.10 (m, 3H), 2.35 (s, 6H).
^{13}C{^{1}H} NMR (151 MHz, $CDCl_3$)	δ 152.9, 151.6, 131.2, 130.7, 129.2, 128.4, 128.2, 122.7, 18.9.

1-(3,5-Dimethylphenyl)-2-phenyldiazene (1l)
3,5-Dimethylaniline (**2l**, 336 mg, 2.72 mmol, 98%) was dissolved in acetic acid (10 mL). Nitrosobenzene (**3**, 300 mg, 2.72 mmol, 97%) was dissolved in acetic acid (10 mL). The reaction was performed following the general procedure and the output was collected for 60 min. The crude product was purified by flash column chromatography (SiO_2, cyclohexane/toluene, 1:1). AB **1l** was obtained as a red oil in a yield of 65% (222 mg, 1.06 mmol, contains <1% azoxybenzene).

Analytical data corresponds to literature.[8]

^{1}H NMR (400 MHz, $CDCl_3$)	δ 7.96 – 7.89 (m, 2H), 7.59 – 7.46 (m, 6H), 7.18 – 7.11 (m, 1H), 2.44 (s, 6H).
^{13}C{^{1}H} NMR (101 MHz, $CDCl_3$)	δ 153.0, 152.9, 138.9, 132.8, 130.9, 129.2, 122.9, 120.8, 21.4.

1-Mesityl-2-phenyldiazene (1m)
2,4,6-Trimethylaniline (**2m**, 379 mg, 2.72 mmol, 97%) was dissolved in acetic acid (10 mL). Nitrosobenzene (**3**, 300 mg, 2.72 mmol, 97%) was dissolved in acetic acid (10 mL). The reaction was performed following the general procedure and the output was collected for 60 min. AB **1m** was obtained as a red oil in a yield of 70% (255 mg, 1.14 mmol, contains <4% azoxybenzene).

Analytical data corresponds to literature.[7]

^{1}H NMR (400 MHz, $CDCl_3$) δ 7.92 – 7.86 (m, 2H), 7.57 – 7.44 (m, 3H), 6.96 (dd, *J* = 1.4, 0.7 Hz, 2H), 2.39 (s, 6H), 2.34 (s, 3H).

$^{13}C\{^1H\}$ NMR (101 MHz, $CDCl_3$) δ 153.1, 149.0, 138.5, 131.5, 130.8, 130.1, 129.2, 122.6, 21.2, 19.3.

1-(3,5-Di-*tert*-butylphenyl)-2-phenyldiazene (1n)
3,5-Di-*tert*-butylaniline (**2n**, 570 mg, 2.72 mmol, 98%) was dissolved in acetic acid (10 mL). Nitrosobenzene (**3**, 300 mg, 2.72 mmol, 97%) was dissolved in acetic acid (10 mL). The reaction was performed following the general procedure and the output was collected for 60 min. AB **1n** was obtained as an orange resin in a yield of 99% (476 mg, 1.62 mmol, contains <1% azoxybenzene).

^{1}H NMR (400 MHz, $CDCl_3$) δ 7.95 – 7.90 (m, 2H, CH_{Ar}), 7.80 (d, *J* = 1.8 Hz, 2H, CH_{Ar}), 7.59 (t, *J* = 1.8 Hz, 1H, CH_{Ar}), 7.55 – 7.5 (m, 2H, CH_{Ar}), 7.49 – 7.44 (m, 1H, CH_{Ar}), 1.42 (s, 18H, CH_3).

$^{13}C\{^1H\}$ NMR (101 MHz, $CDCl_3$) δ 153.1, 152.8, 152.0, 130.7, 129.2, 125.5, 122.8, 117.4, 35.3, 31.6.

HRMS (ESI): calc. for $[C_{20}H_{26}N_2Na]^+$: $[M+Na]^+$ 317.1988, found 317.1984.

4-(2-Phenyldiazenyl)benzonitrile (1o)
4-Aminobenzonitrile (**2o**, 328 mg, 2.72 mmol, 98%) was dissolved in acetic acid (10 mL). Nitrosobenzene (**3**, 300 mg, 2.72 mmol, 97%) was dissolved in acetic acid (10 mL). The reaction was performed following the general procedure and the output was collected for 60 min. AB **1o** was obtained as an orange solid in a yield of 7% (25 mg, 0.12 mmol, contains <2% azoxybenzene).

Analytical data corresponds to literature.[9]

^{1}H NMR (400 MHz, $CDCl_3$) δ 8.03 – 7.92 (m, 4H), 7.85 – 7.79 (m, 2H), 7.59 – 7.51 (m, 3H).

$^{13}C\{^1H\}$ NMR (151 MHz, $CDCl_3$) δ 154.6, 152.5, 133.4, 132.4, 129.4, 123.5, 123.5, 118.6, 114.1.

The synthesis was repeated at 90 °C and 110 °C. A back pressure regulator (75 psi, 5.2 bar) was added in case of the synthesis at 110 °C. After column chromatography (SiO_2, cyclohexane/toluene 1:1), AB **1o** was obtained in a yield of 17% (90 °C, 57 mg, 0.28 mmol) and 19% (110 °C, 64 mg, 0.31 mmol) respectively.

3-(2-Phenyldiazenyl)benzonitrile (1p)
3-Aminobenzonitrile (**2p**, 328 mg, 2.72 mmol, 98%) was dissolved in acetic acid (10 mL). Nitrosobenzene (**3**, 300 mg, 2.72 mmol, 97%) was dissolved in acetic acid (10 mL). The reaction was performed following the general procedure and the output was collected for 60 min. AB **1p** was obtained as an orange solid in a yield of 54% (183 mg, 0.88 mmol, contains <4% azoxybenzene).

Analytical data corresponds to literature.[10, 11]

^{1}H NMR (400 MHz, $CDCl_3$) δ 8.21 – 8.18 (m, 1H), 8.15 (ddd, *J* = 8.0, 2.0, 1.2 Hz, 1H), 7.98 – 7.93 (m, 2H), 7.74 (dt, *J* = 7.7, 1.4 Hz, 1H), 7.68 – 7.60 (m, 1H), 7.57 – 7.52 (m, 3H).

$^{13}C\{^1H\}$ NMR (101 MHz, $CDCl_3$) δ 152.5, 152.2, 133.8, 132.1, 130.1, 129.3, 127.4, 126.2, 123.3, 118.3, 113.4.

4-Phenylazophenol (1q)
p-Aminophenol (**2q**, 300 mg, 2.72 mmol, 99%) was dissolved in acetic acid (10 mL). Nitrosobenzene (**3**, 300 mg, 2.72 mmol, 97%) was dissolved in acetic acid (10 mL). The reaction was performed following the general procedure and the output was collected for 60 min. AB **1q** was obtained as a yellow solid in a yield of 68% (221 mg, 1.11 mmol, contains <1% azoxybenzene).

Analytical data corresponds to literature.[12]

^{1}H NMR (400 MHz, $CDCl_3$)	δ 7.93 – 7.85 (m, 4H), 7.54 – 7.48 (m, 2H), 7.47 – 7.42 (m, 1H), 6.97 – 6.92 (m, 2H), 5.30 (s, 1H).
^{13}C{^{1}H} NMR (101 MHz, $CDCl_3$)	δ 158.4, 152.8, 147.3, 130.6, 129.2, 125.2, 122.7, 116.0.

1-Phenyl-2-(4-(trifluoromethyl)phenyl)diazene (1r)
4-(Trifluoromethyl)aniline (**2r**, 452 mg, 2.72 mmol, 97%) was dissolved in acetic acid (10 mL). Nitrosobenzene (**3**, 300 mg, 2.72 mmol, 97%) was dissolved in acetic acid (10 mL). The reaction was performed following the general procedure and the output was collected for 60 min. The crude product was purified by flash column chromatography (SiO_2, cyclohexane/ethyl acetate, 0–10%). AB **1r** was obtained as an orange solid in a yield of 33% (135 mg, 0.540 mmol, contains <3% azoxybenzene).

Analytical data corresponds to literature.[4]

^{1}H NMR (400 MHz, $CDCl_3$)	δ 8.01 (dt, *J* = 8.1, 0.9 Hz, 2H), 7.98 – 7.93 (m, 2H), 7.82 – 7.75 (m, 2H), 7.58 – 7.51 (m, 3H).
^{13}C{^{1}H} NMR (101 MHz, $CDCl_3$)	δ 154.6, 154.6, 152.6, 132.4 (d, $^2J_{CF}$ = 32.4 Hz), 132.0, 129.3, 126.4 (q, $^3J_{CF}$ = 3.7 Hz) , 125.4, 123.3, 123.2.

1-(4-Fluorophenyl)-2-phenyldiazene (1s)
4-Fluoroaniline (**2s**, 305 mg, 2.72 mmol, 99%) was dissolved in acetic acid (10 mL). Nitrosobenzene (**3**, 300 mg, 2.72 mmol, 97%) was dissolved in acetic acid (10 mL). The reaction was performed following the general procedure and the output was collected for 60 min. AB **1s** was obtained as a yellow solid in a yield of 95% (310 mg, 1.55 mmol, contains <2% azoxybenzene).

Analytical data corresponds to literature.[4]

^{1}H NMR (400 MHz, $CDCl_3$)	δ 8.00 – 7.87 (m, 4H), 7.58 – 7.44 (m, 3H), 7.24 – 7.16 (m, 2H).
^{13}C{^{1}H} NMR (101 MHz, $CDCl_3$)	δ 164.5 (d, $^1J_{CF}$ = 252.0 Hz), 152.63, 149.3 (d, $^4J_{CF}$ = 3.0 Hz), 131.2, 129.3, 125.0 (d, $^3J_{CF}$ = 9.0Hz), 123.0, 116.2 (d, $^2J_{CF}$ = 22.9 Hz).

Large scale synthesis of 1t

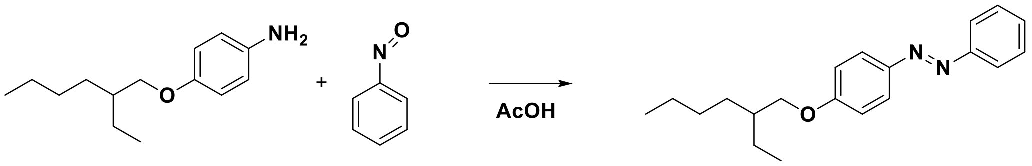

2t **3** **1t**

Aniline **2t** and nitrosobenzene (**3**) were each dissolved in acetic acid (99%, c = 272 mmol/L, respectively) in separate bottles and degassed by a nitrogen stream for 60 min. Additionally, a third bottle with cyclohexane was prepared. Both starting material solutions were pumped individually at 0.2 mL/min. The streams were mixed in a T-mixer and fed into a tubular reactor (20 mL, 50 min residence time) at 70 °C. After passing the reactor, the reaction mixture was diluted by cyclohexane (2.0 mL/min). After the calculated residence time and a short equilibration time, the output was collected for 72 h. The reaction mixture was fed into brine (500 mL) in a separatory funnel (analogue to Figure S1). After intervals of 12 h, the organic layer was separated, dried with $MgSO_4$, and the solvent was removed under reduced pressure. The solvent (cyclohexane) was recycled to reduce the overall solvent consumption. AB **1t** was obtained as red oil in a yield of >99% (72.4 g, 233 mmol, contains <4% azoxybenzene).

Analytical data corresponds to literature.[13]

^{1}H NMR (400 MHz, $CDCl_3$)	δ 7.96 – 7.84 (m, 4H), 7.55 – 7.46 (m, 2H), 7.48 – 7.39 (m, 1H), 7.06 – 6.98 (m, 2H), 3.99 – 3.88 (m, 2H), 1.76 (h, J = 6.2 Hz, 1H), 1.62 – 1.26 (m, 8H), 0.99 – 0.90 (m, 6H).
^{13}C{^{1}H} NMR (101 MHz, $CDCl_3$)	δ 162.1, 153.0, 147.0, 130.4, 129.2, 124.9, 122.7, 114.9, 71.0, 39.5, 30.7, 29.2, 24.0, 23.2, 14.2, 11.3.

Unsuccessful substrates (did not result in AB formation)

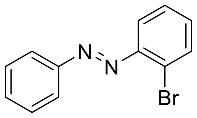

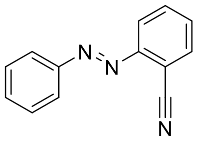

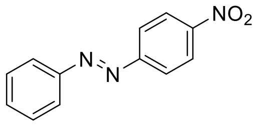

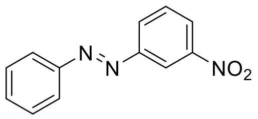

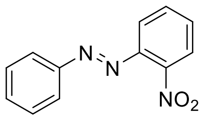

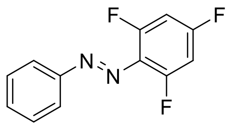

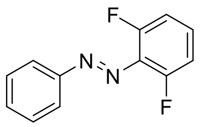

NMR spectra

For all azobenzenes, the thermodynamically more stable (*E*)-isomer is reported and integrated. Additional small peaks can be attributed to small amounts of (*Z*)-isomers. Peaks at 7.40–7.54 ppm and 8.05–8.32 ppm can be attributed to unsubstituted azoxybenzene. The percentage of unsubstituted azoxybenzene is ≤4% in all synthesized compounds **1a**–**t**.[14]

Azobenzene (1a)

^{1}H NMR

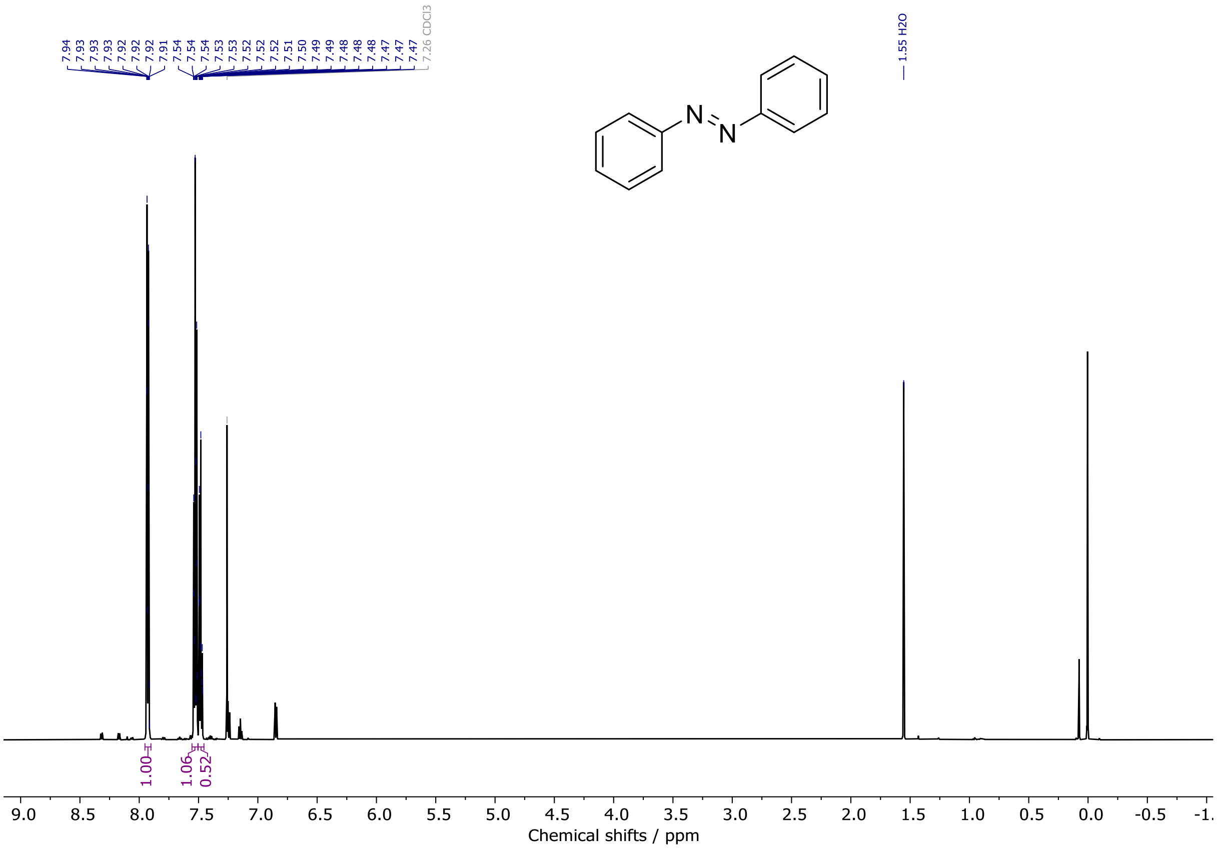

^{13}C NMR

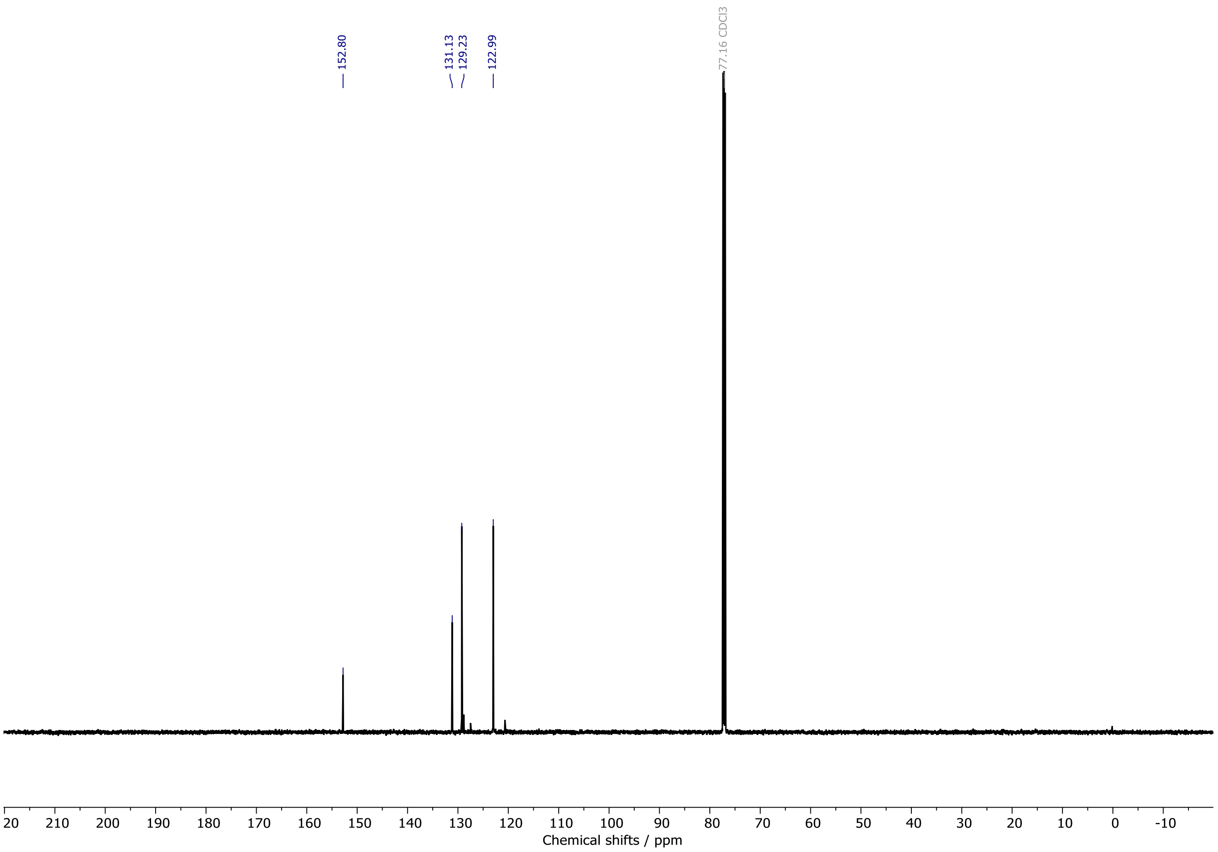

1-(4-Bromophenyl)-2-phenyldiazene (1b)

^{1}H NMR

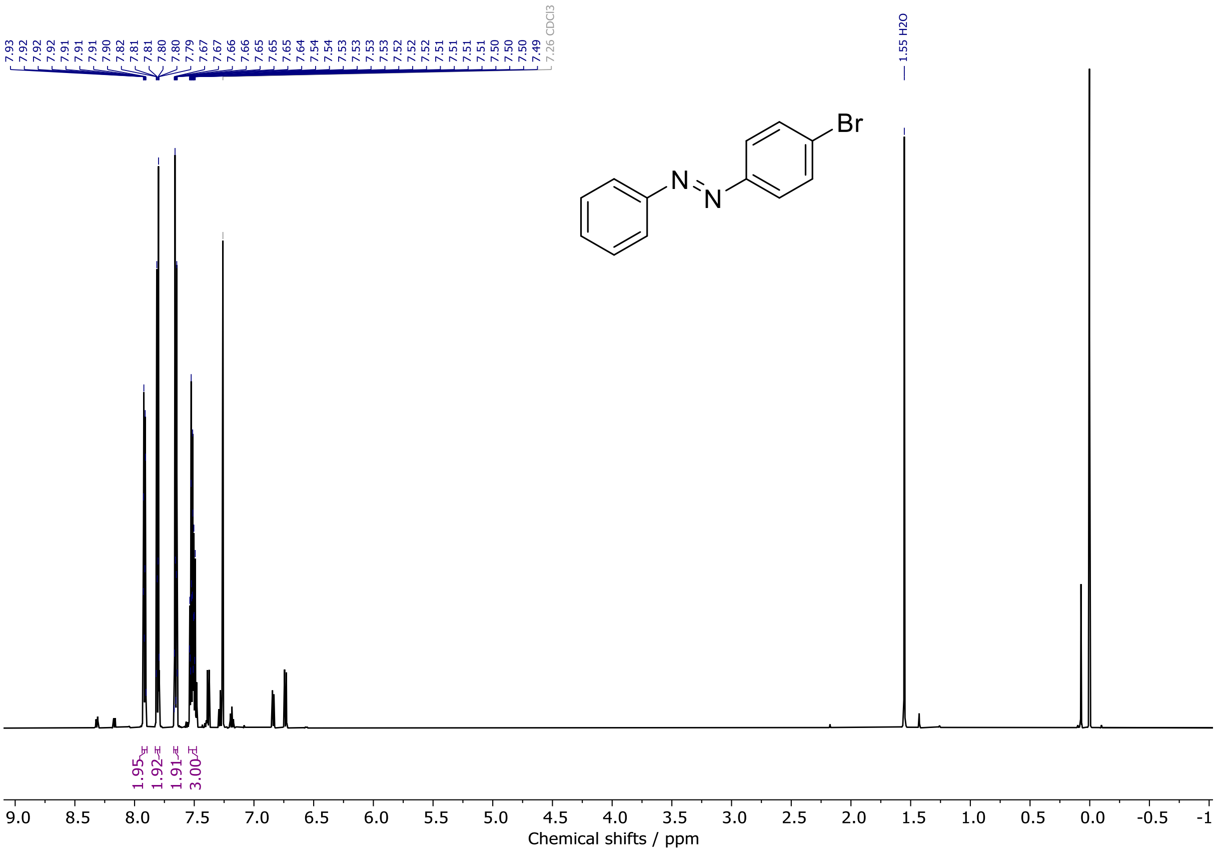

^{13}C NMR

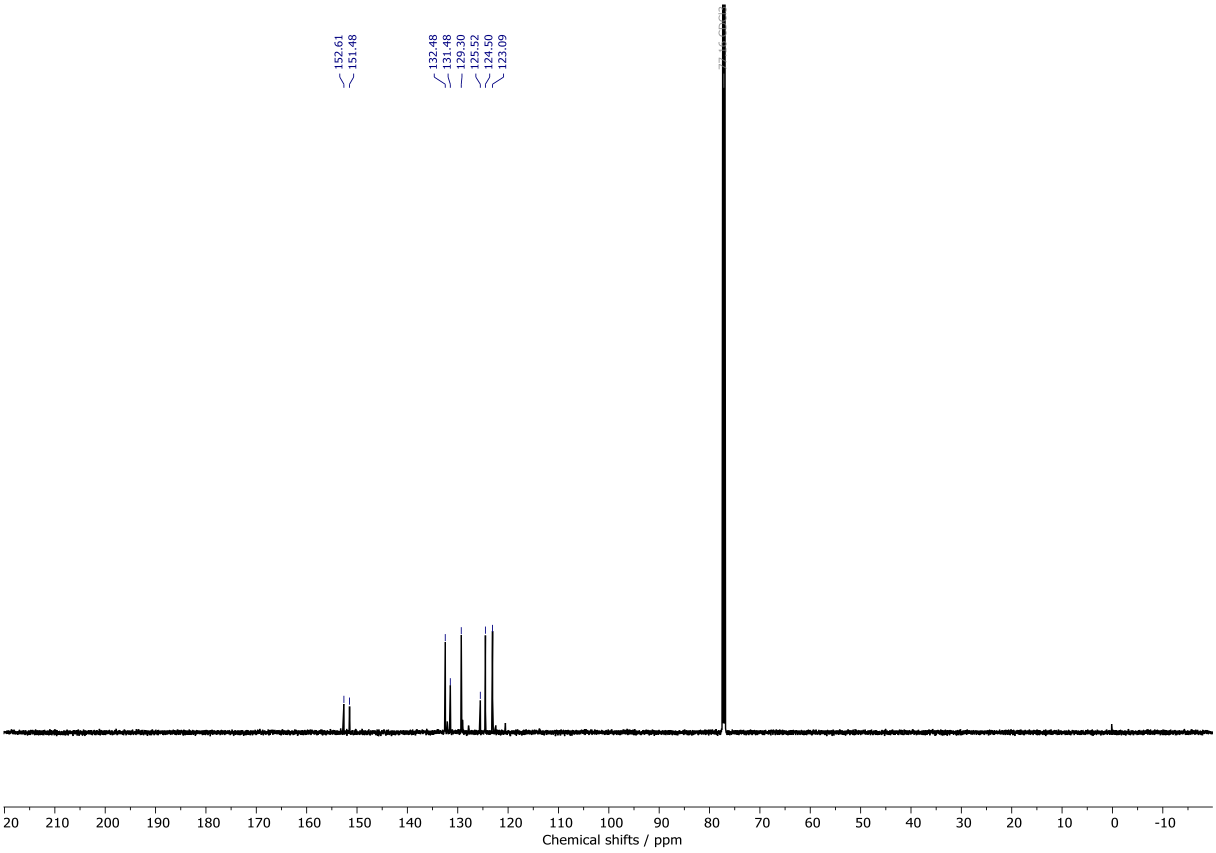

1-(3-Bromophenyl)-2-phenyldiazene (1c)

<u>^{1}H NMR</u>

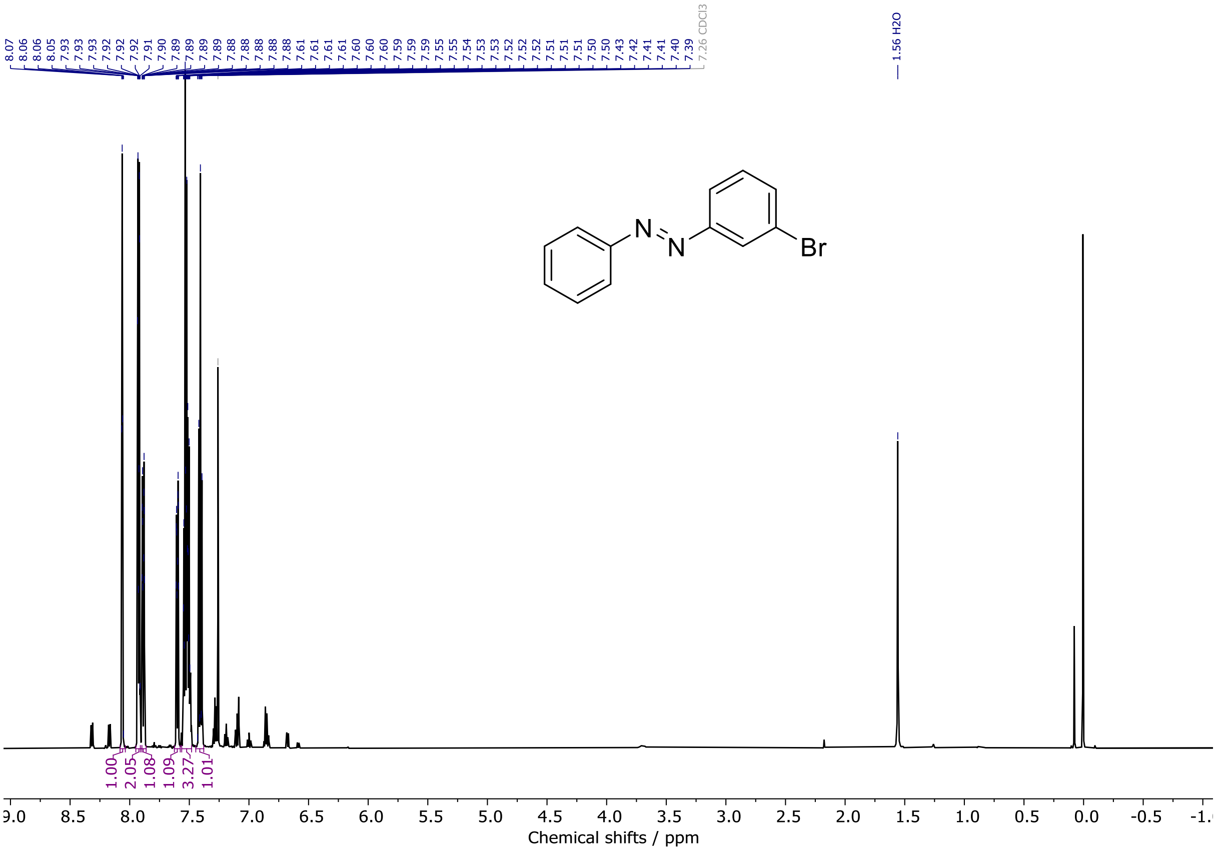

<u>^{13}C NMR</u>

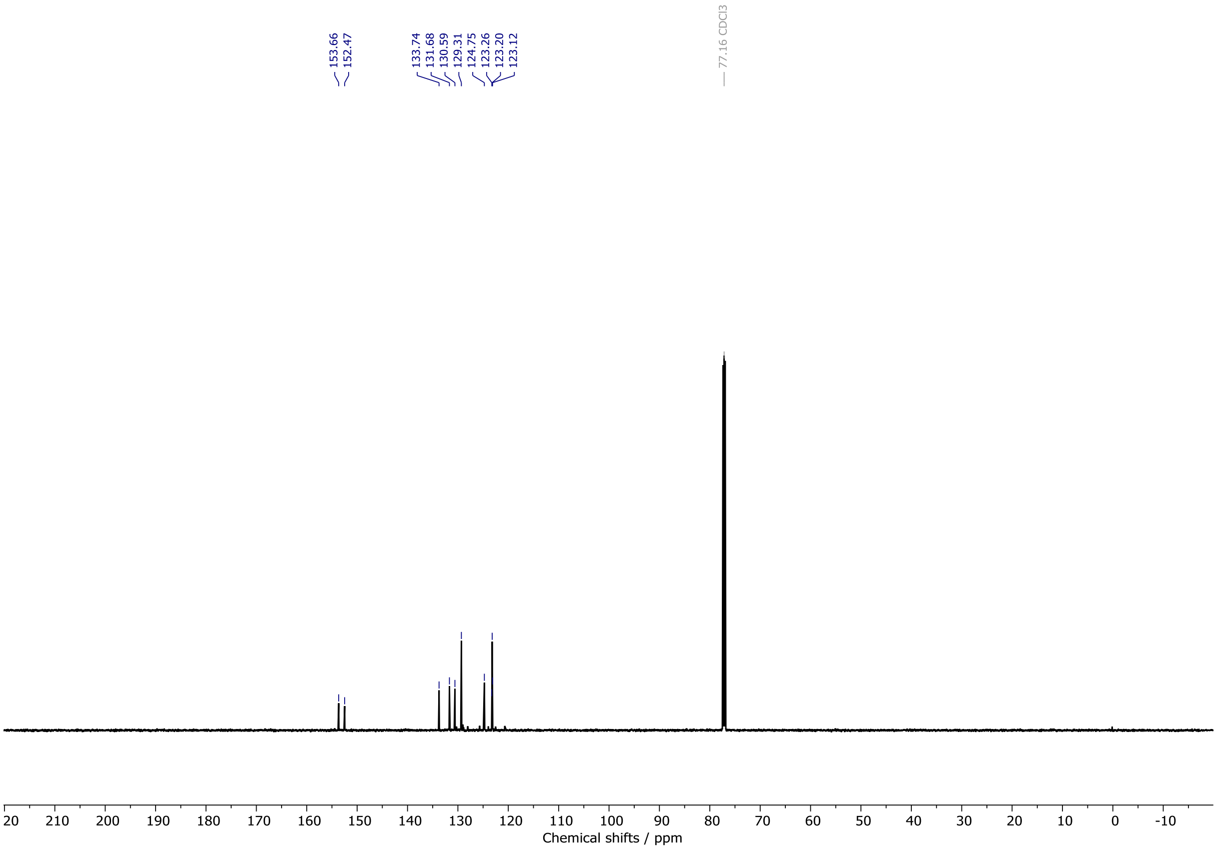

1-(4-Tolyl)-2-phenyldiazene (1d)

<u>^{1}H NMR</u>

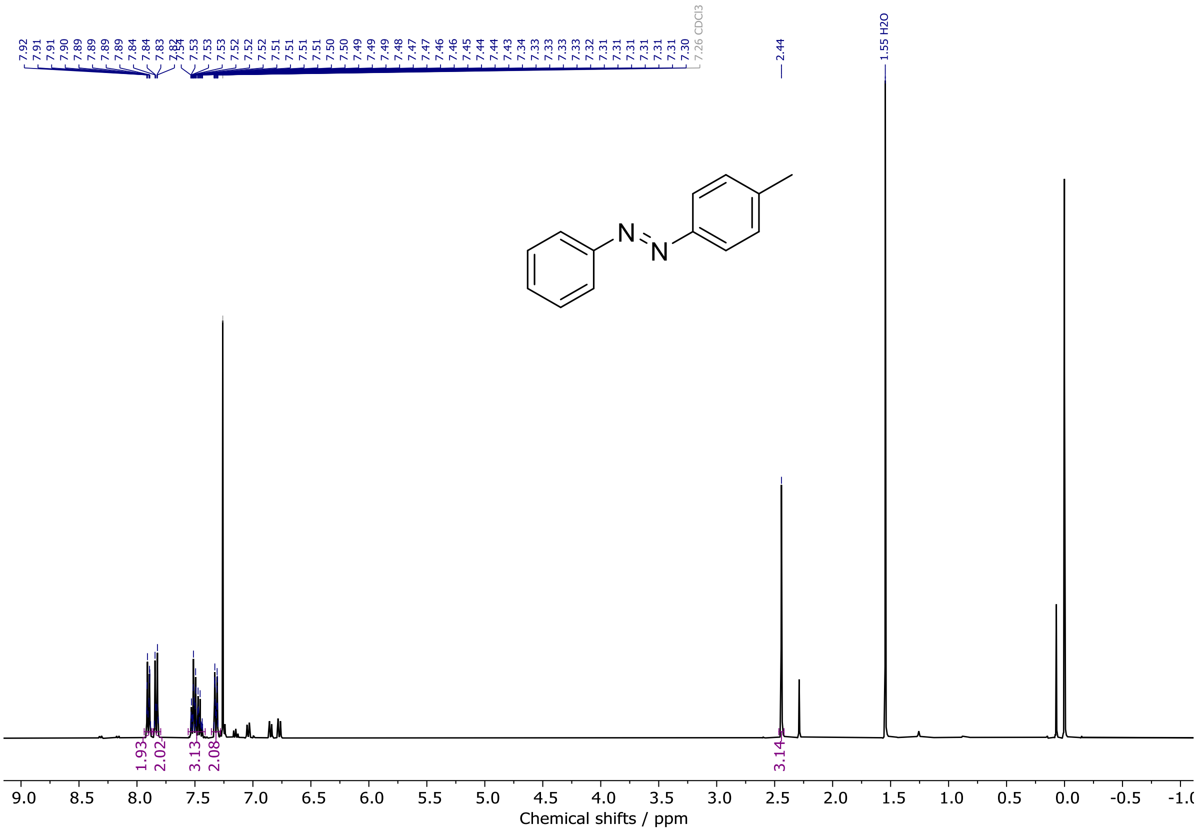

<u>^{13}C NMR</u>

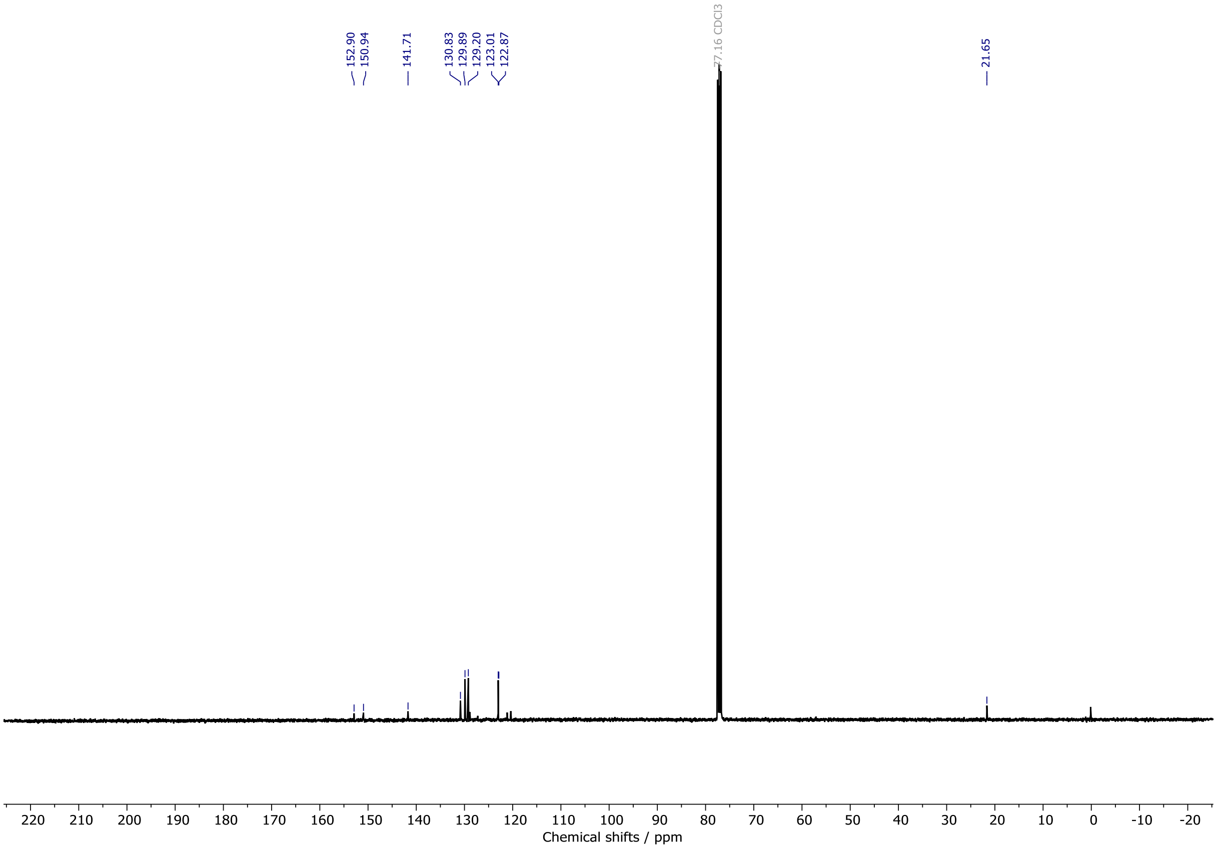

1-(3-Tolyl)-2-phenyldiazene (1e)

^{1}H NMR

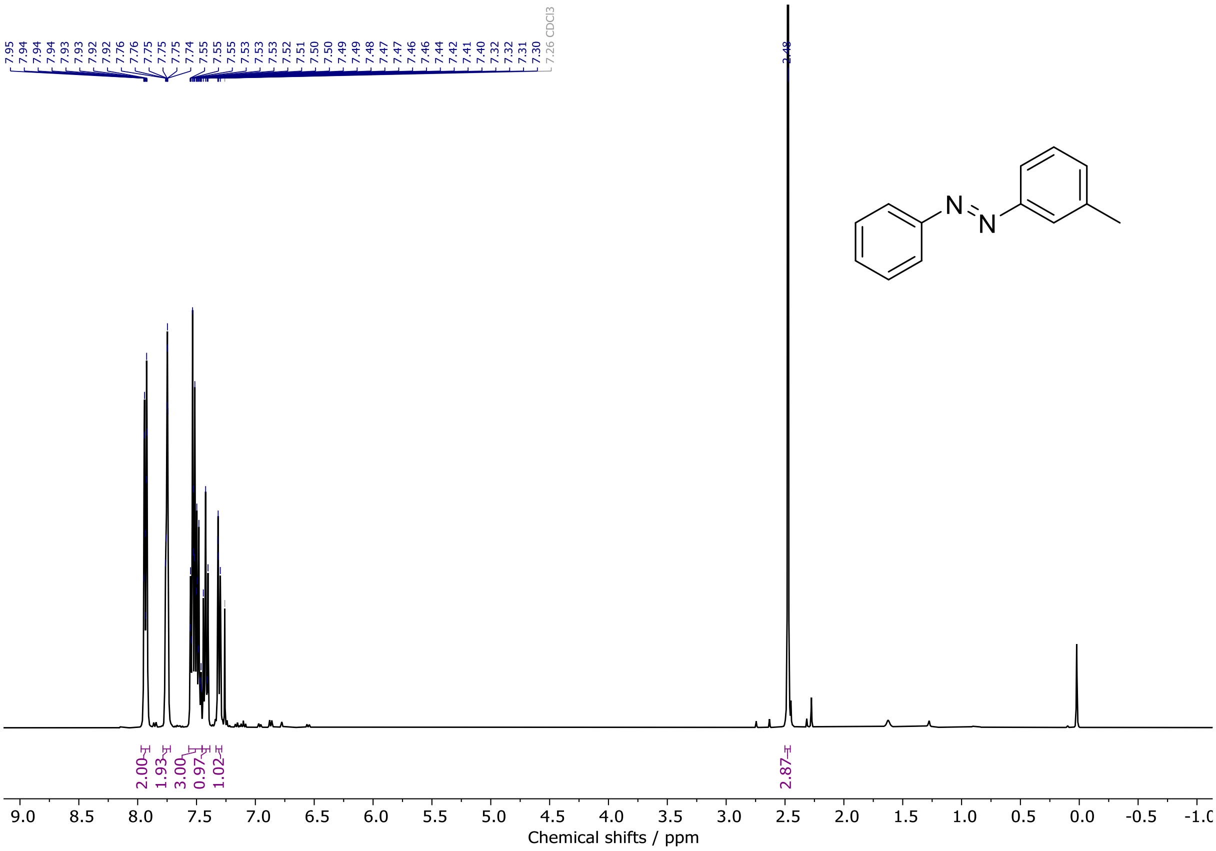

^{13}C NMR

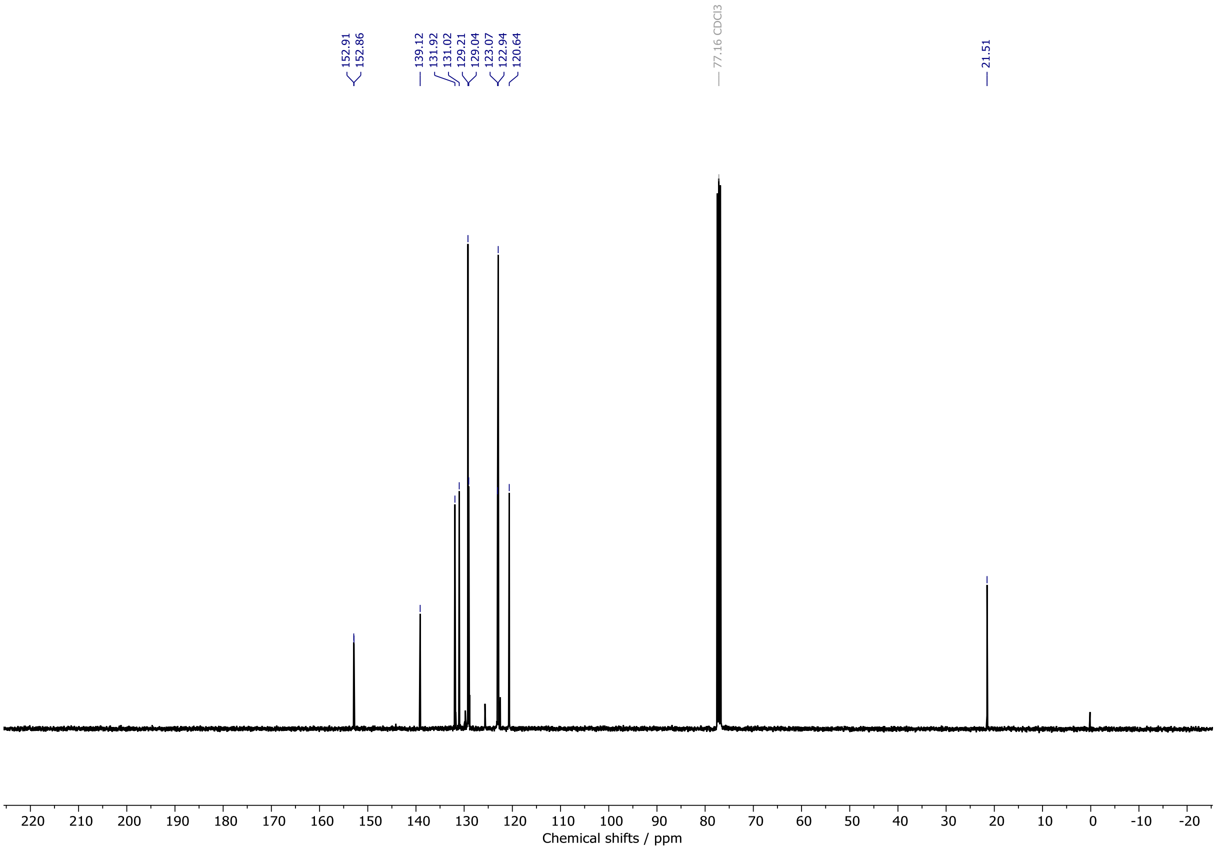

1-Phenyl-2-(*o*-tolyl)diazene (1f)

^{1}H NMR

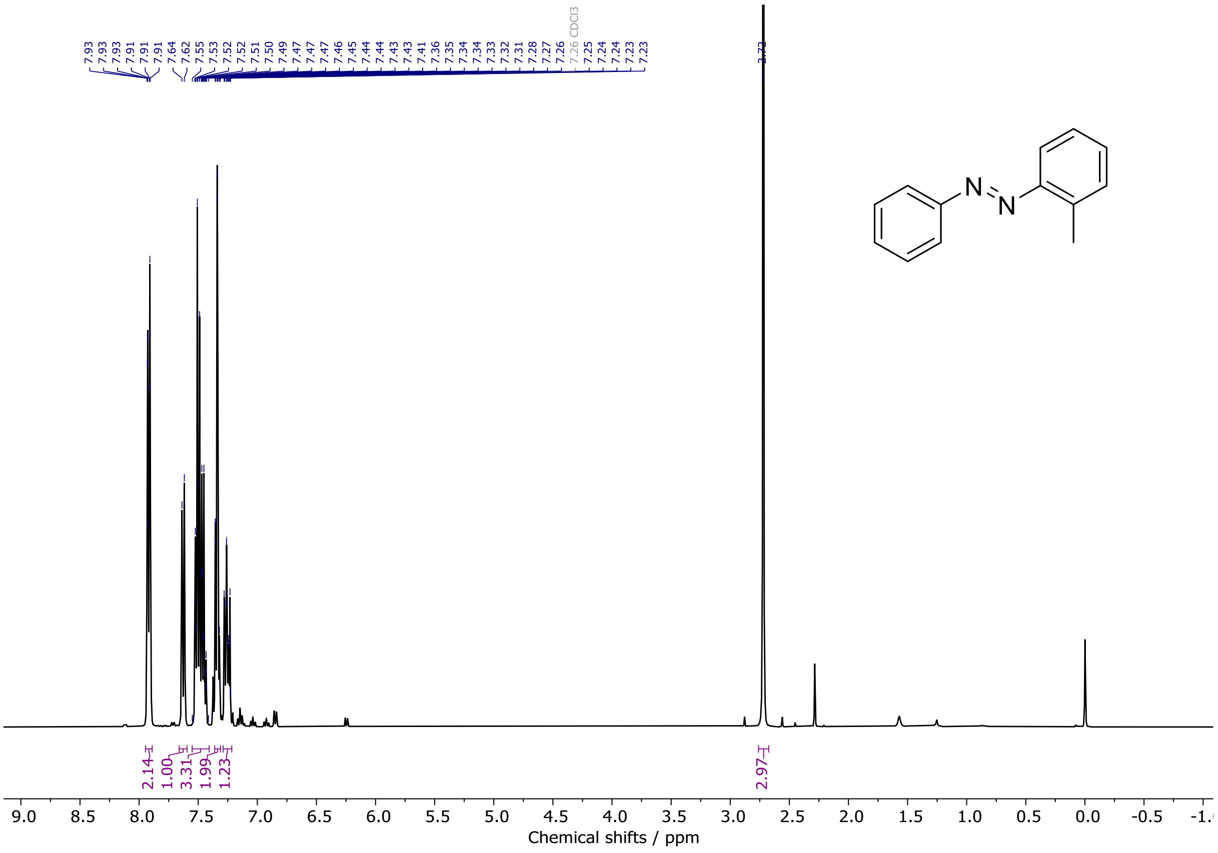

^{13}C NMR

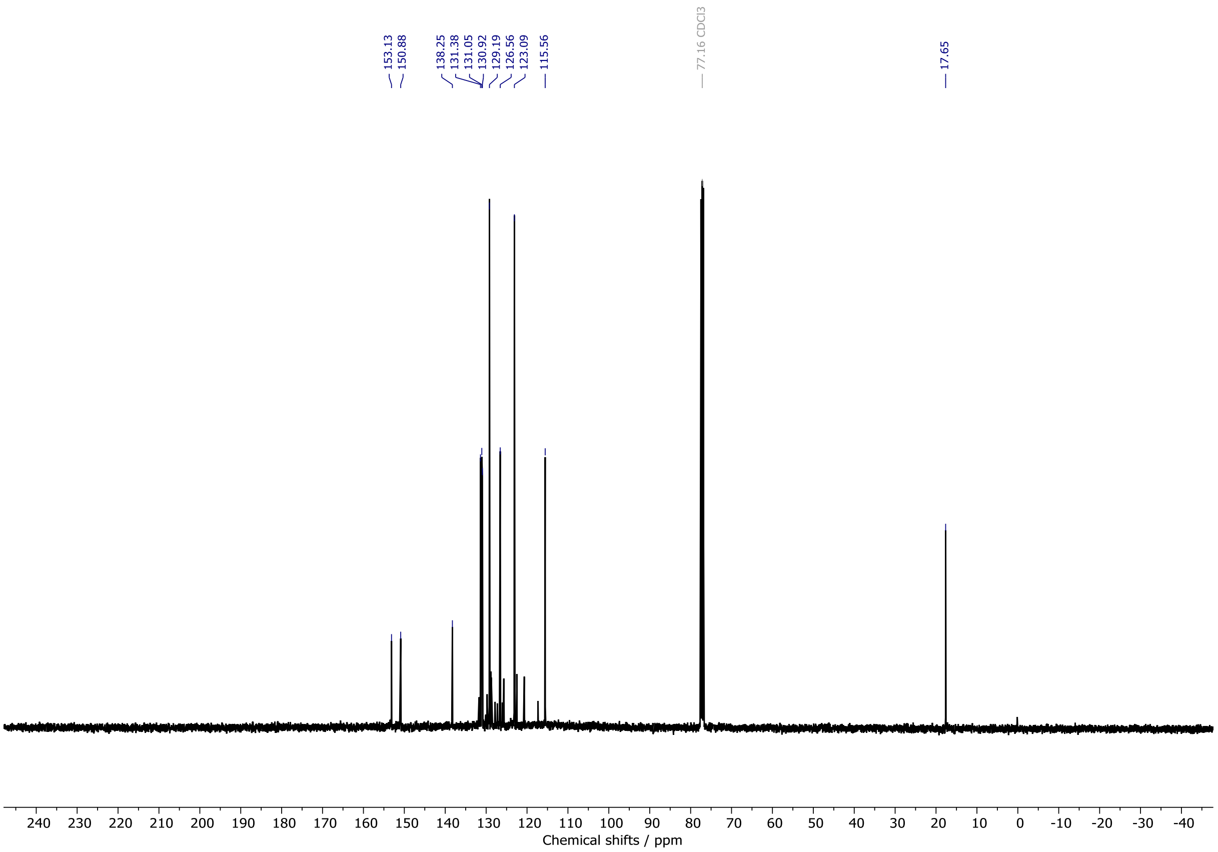

1-(4-(*tert*-Butyl)phenyl)-2-phenyldiazene (1g)

^{1}H NMR

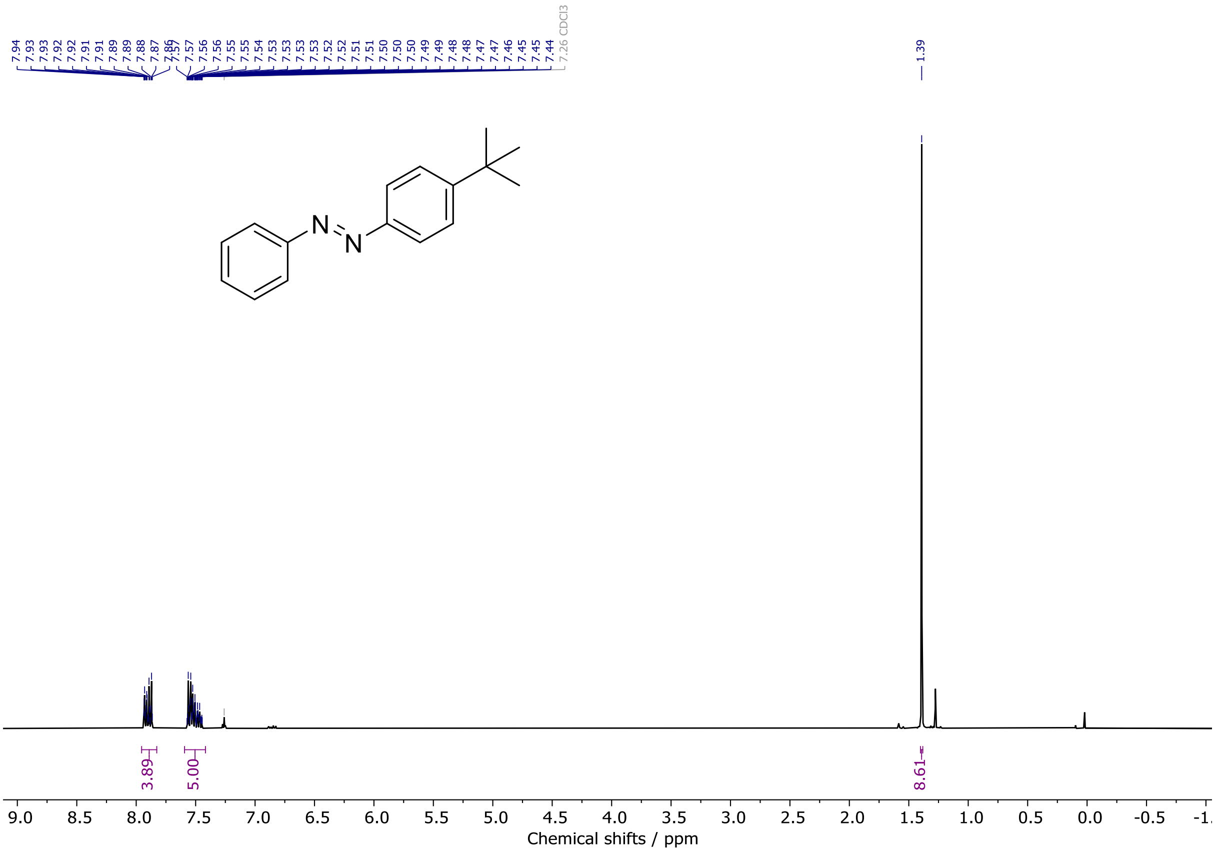

^{13}C NMR

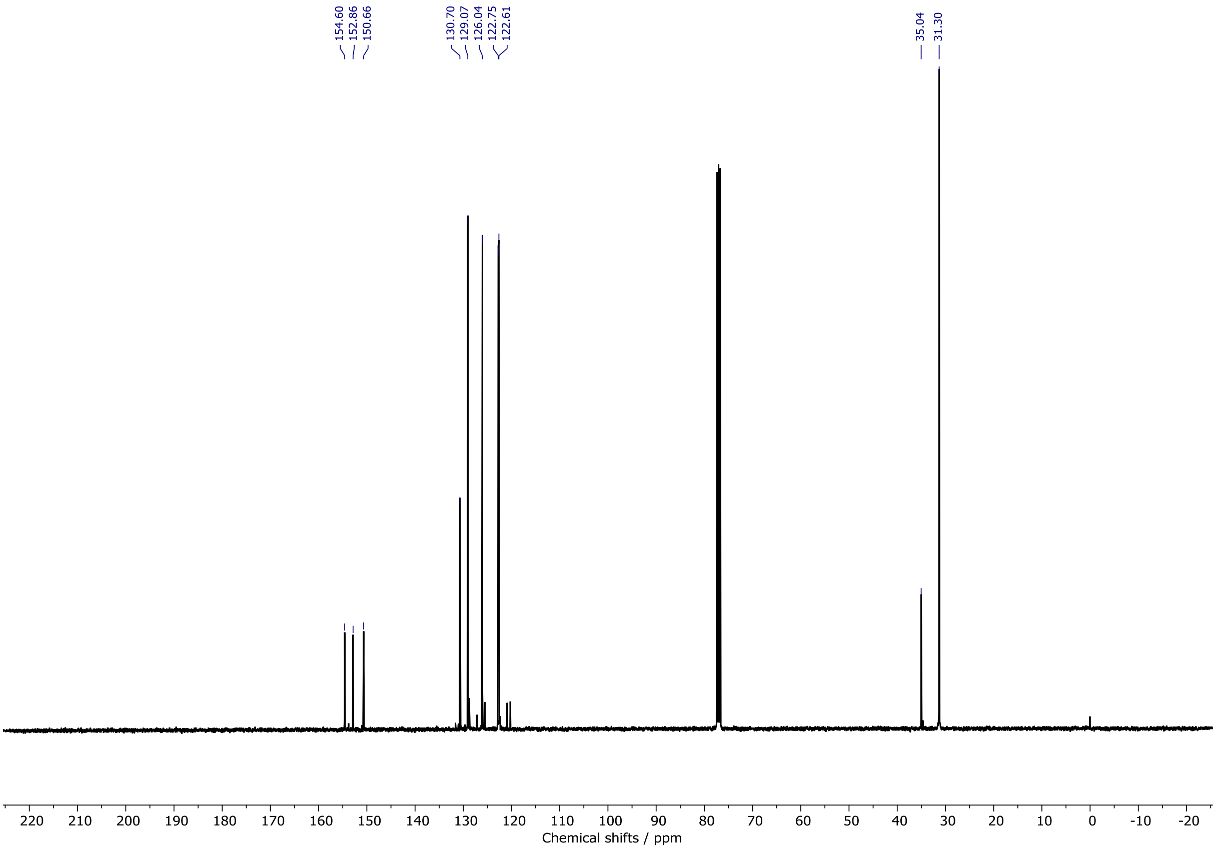

1-(4-Methoxyphenyl)-2-phenyldiazene (1h)

^{1}H NMR

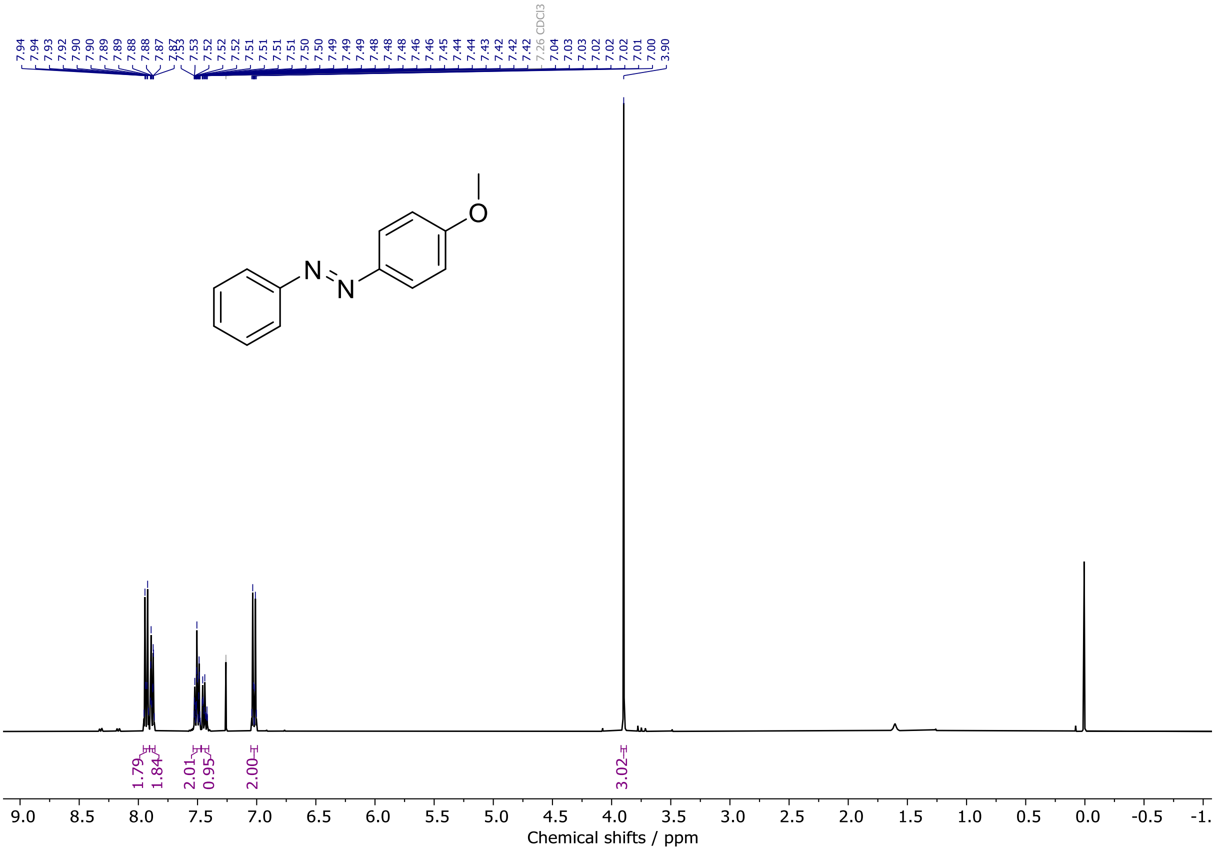

^{13}C NMR

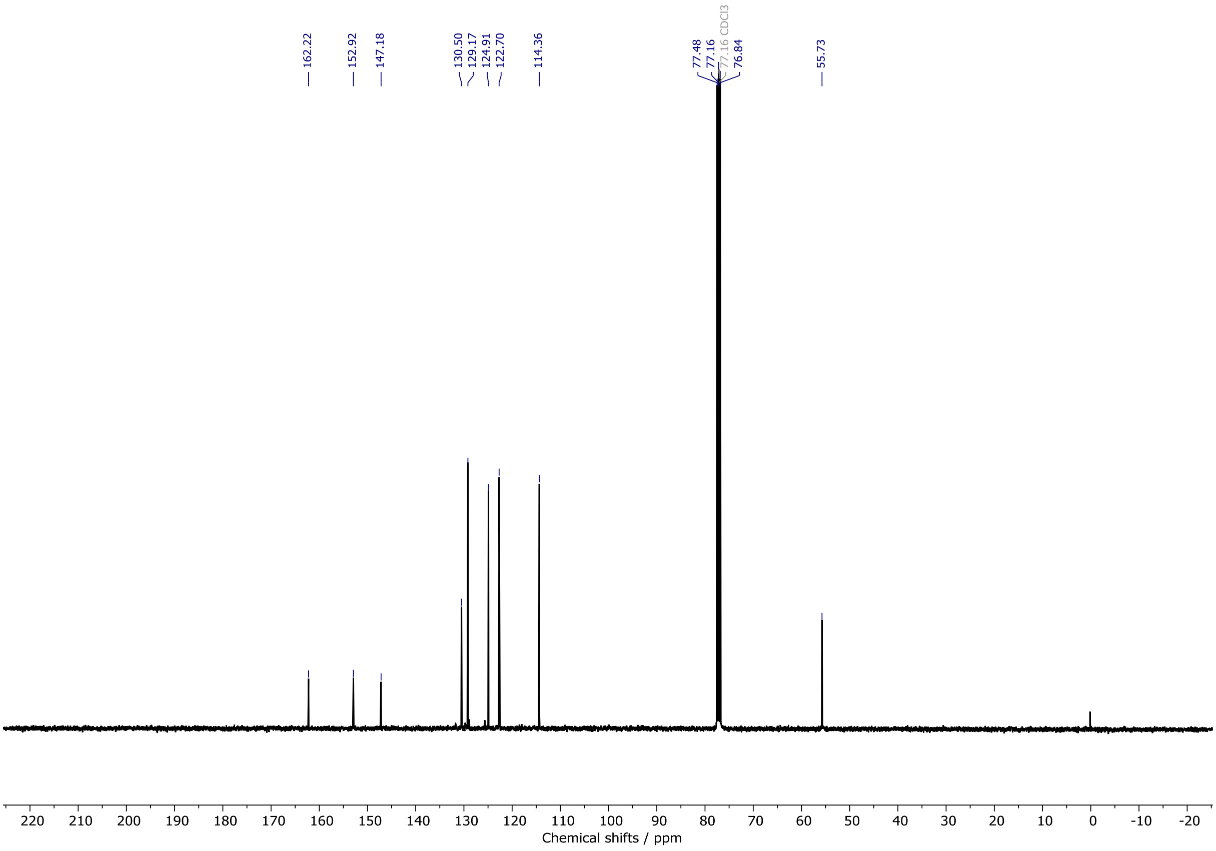

1-(3-Methoxyphenyl)-2-phenyldiazene (1i)

^{1}H NMR

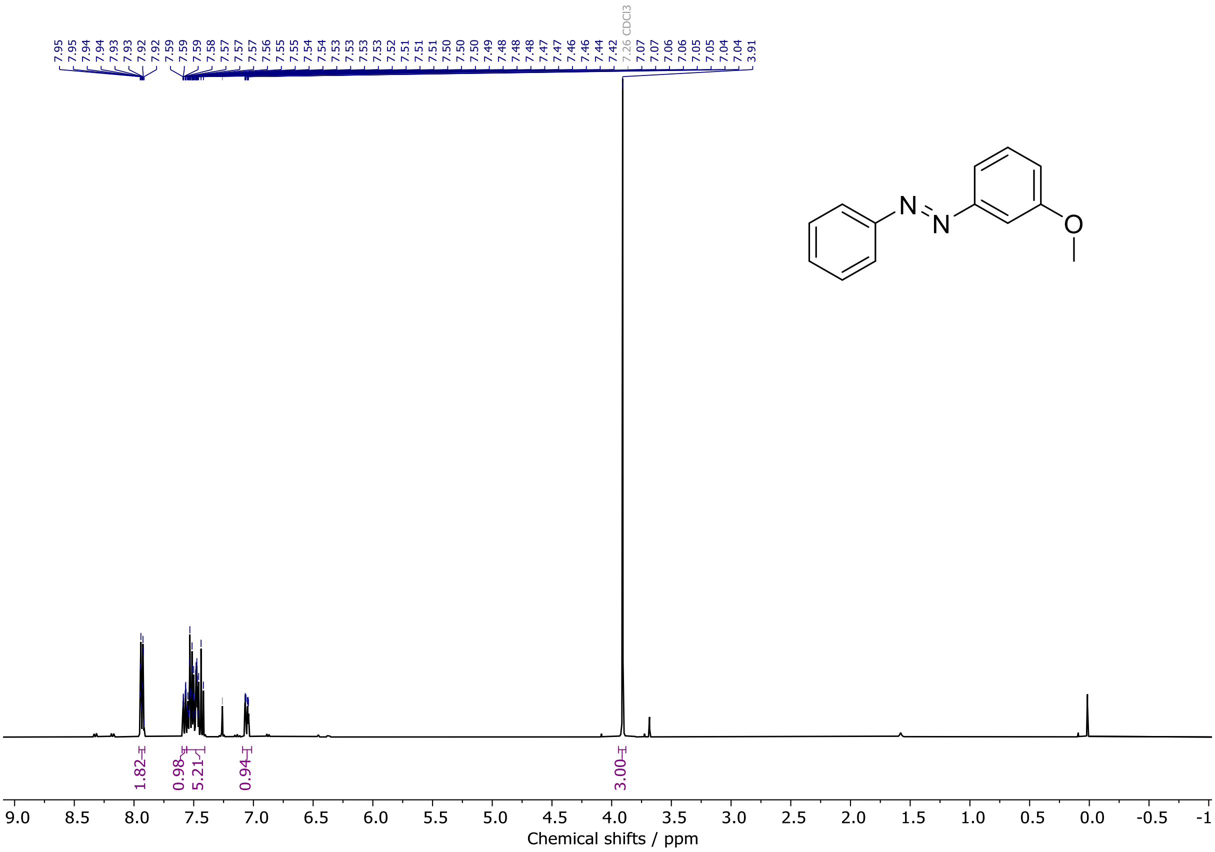

^{13}C NMR

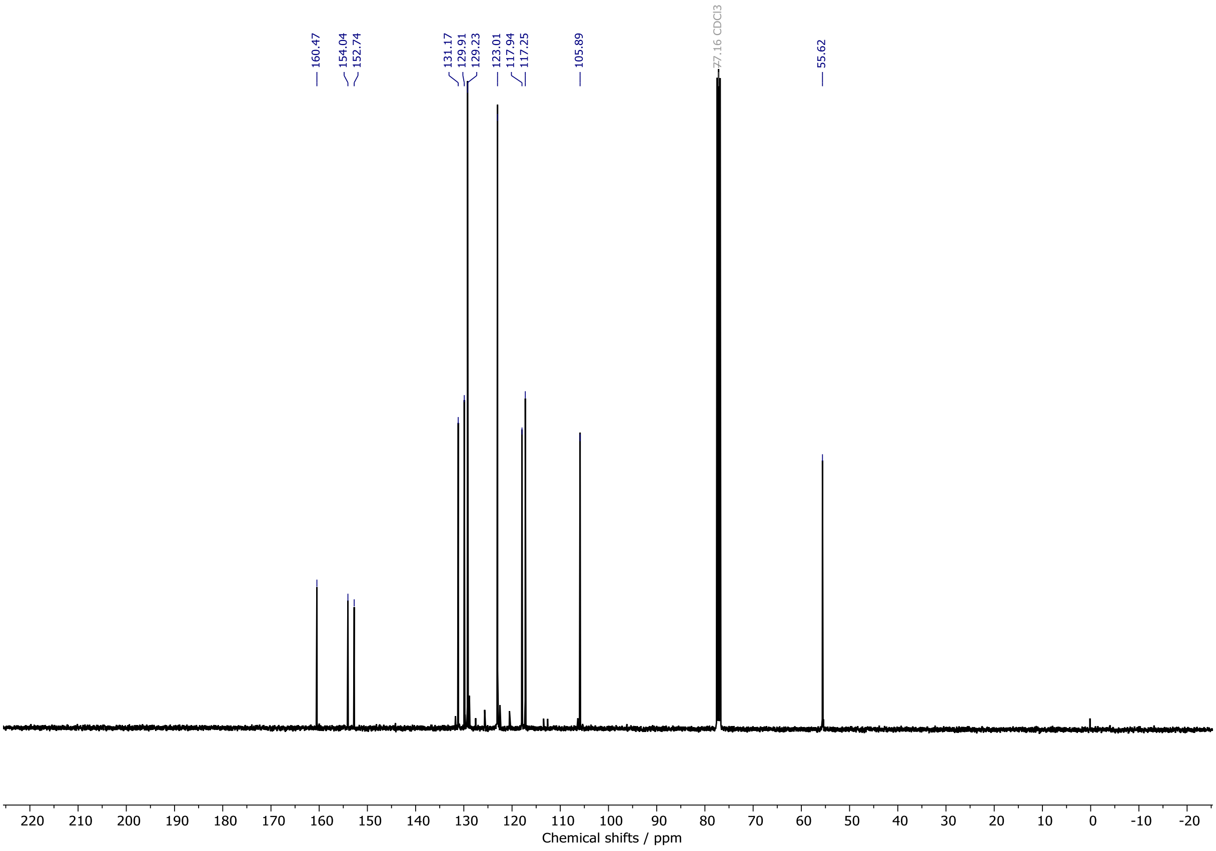

1-(2-Methoxyphenyl)-2-phenyldiazene (1j)

^{1}H NMR

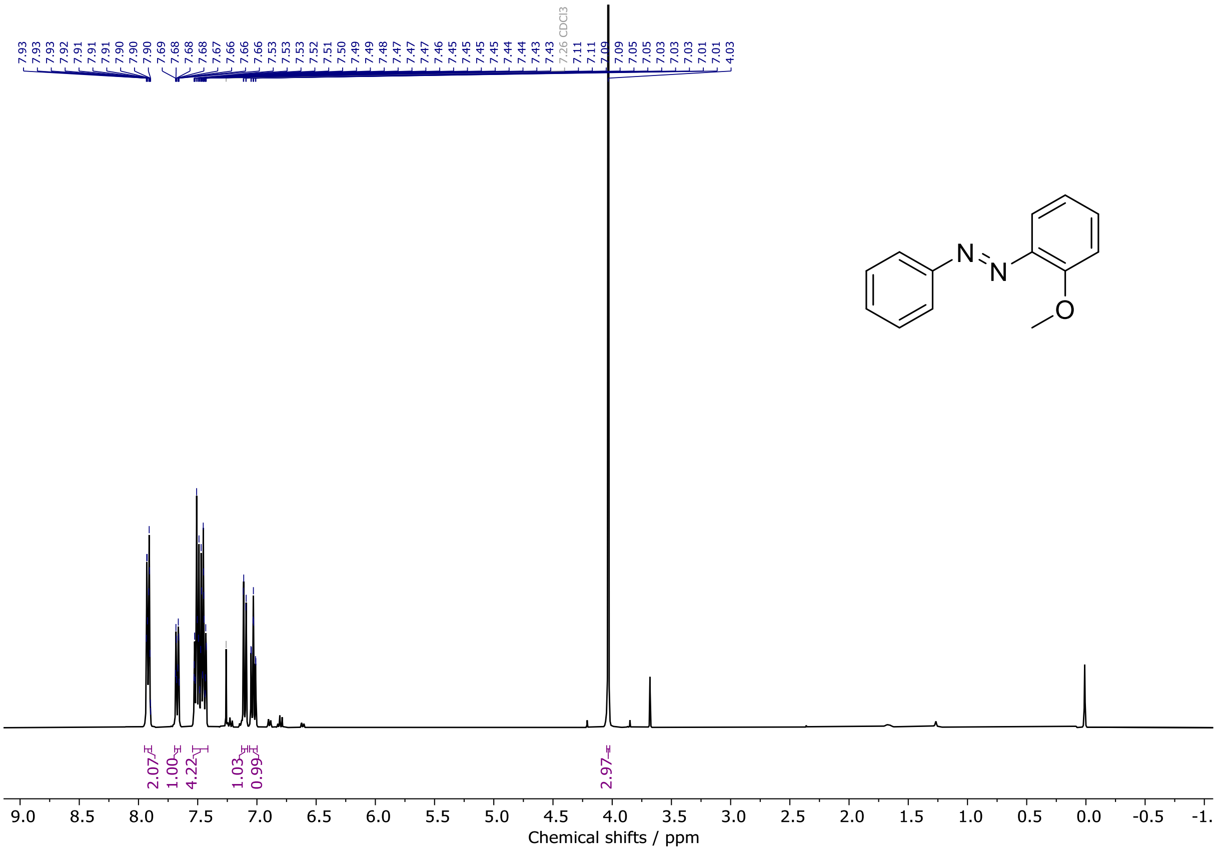

^{13}C NMR

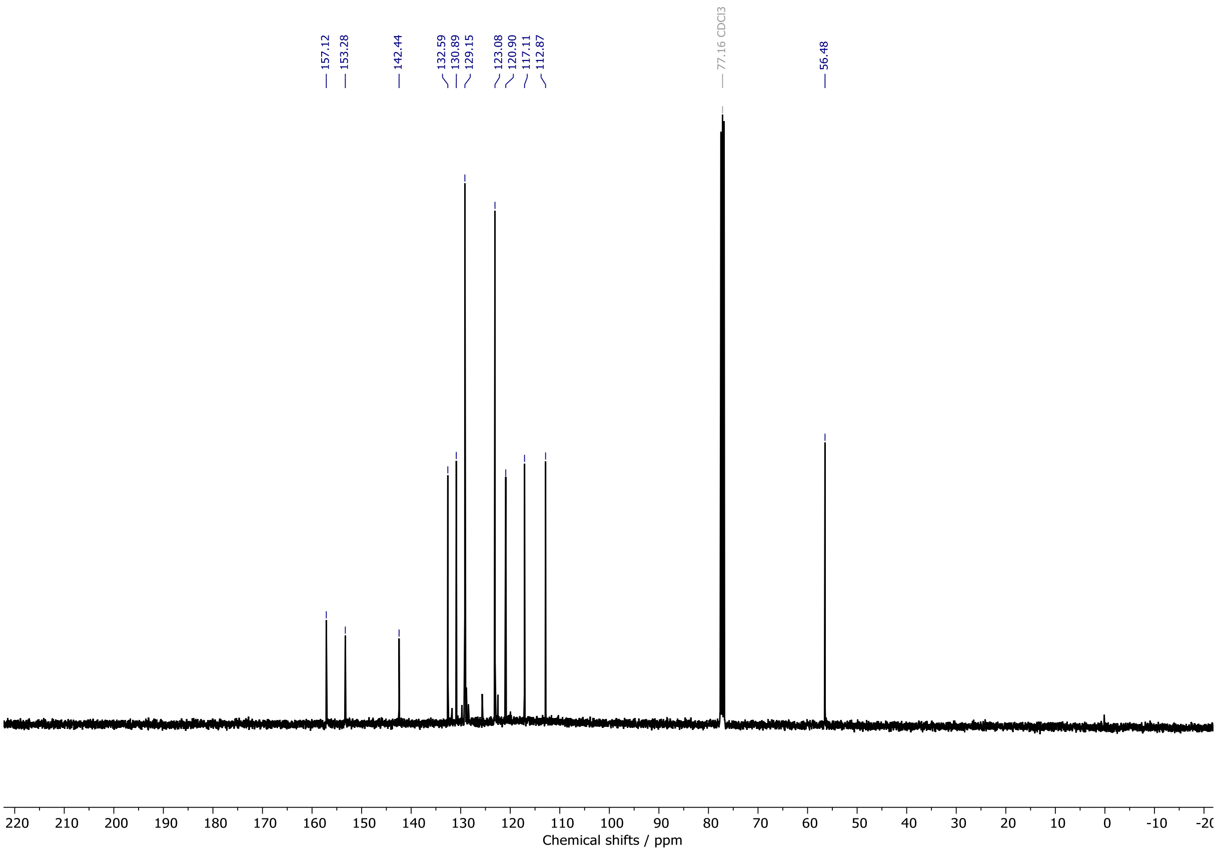

1-(2,6-Dimethylphenyl)-2-phenyldiazene (1k)

^{1}H NMR

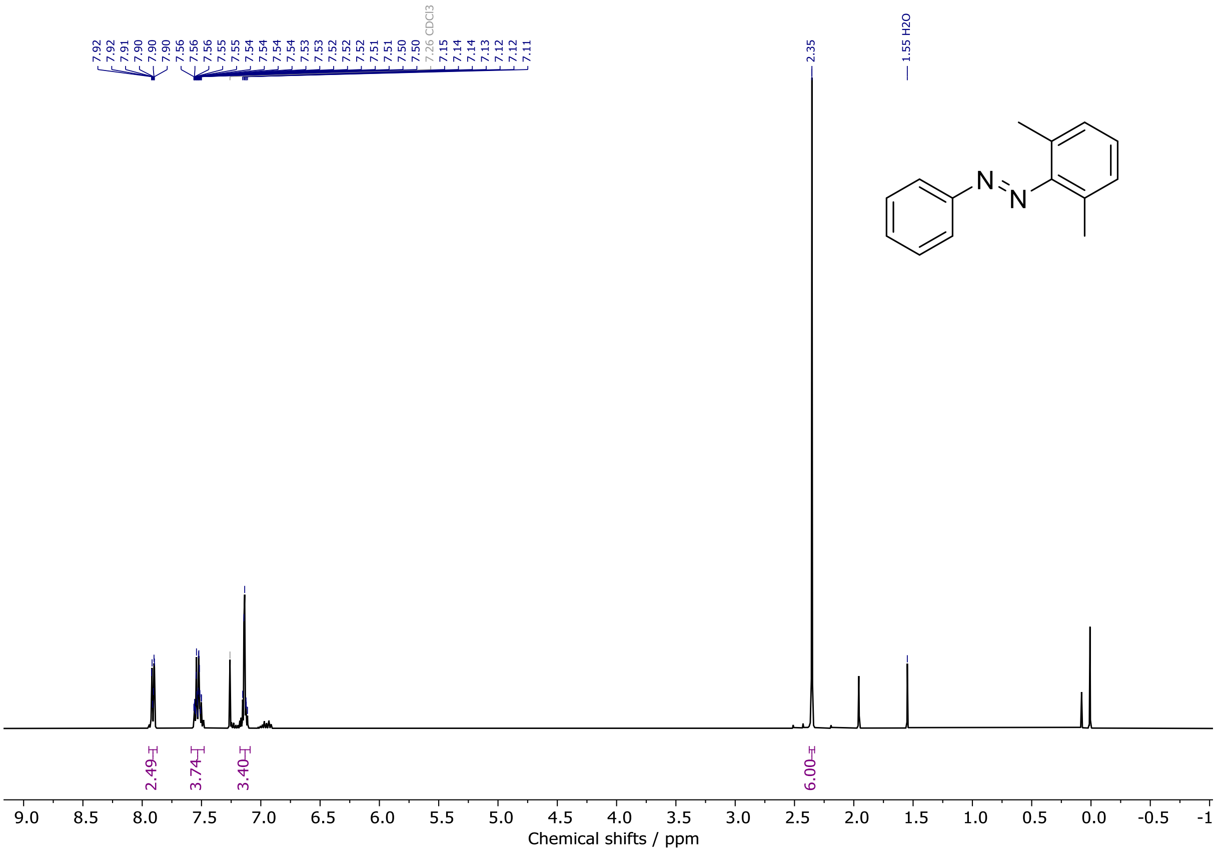

^{13}C NMR

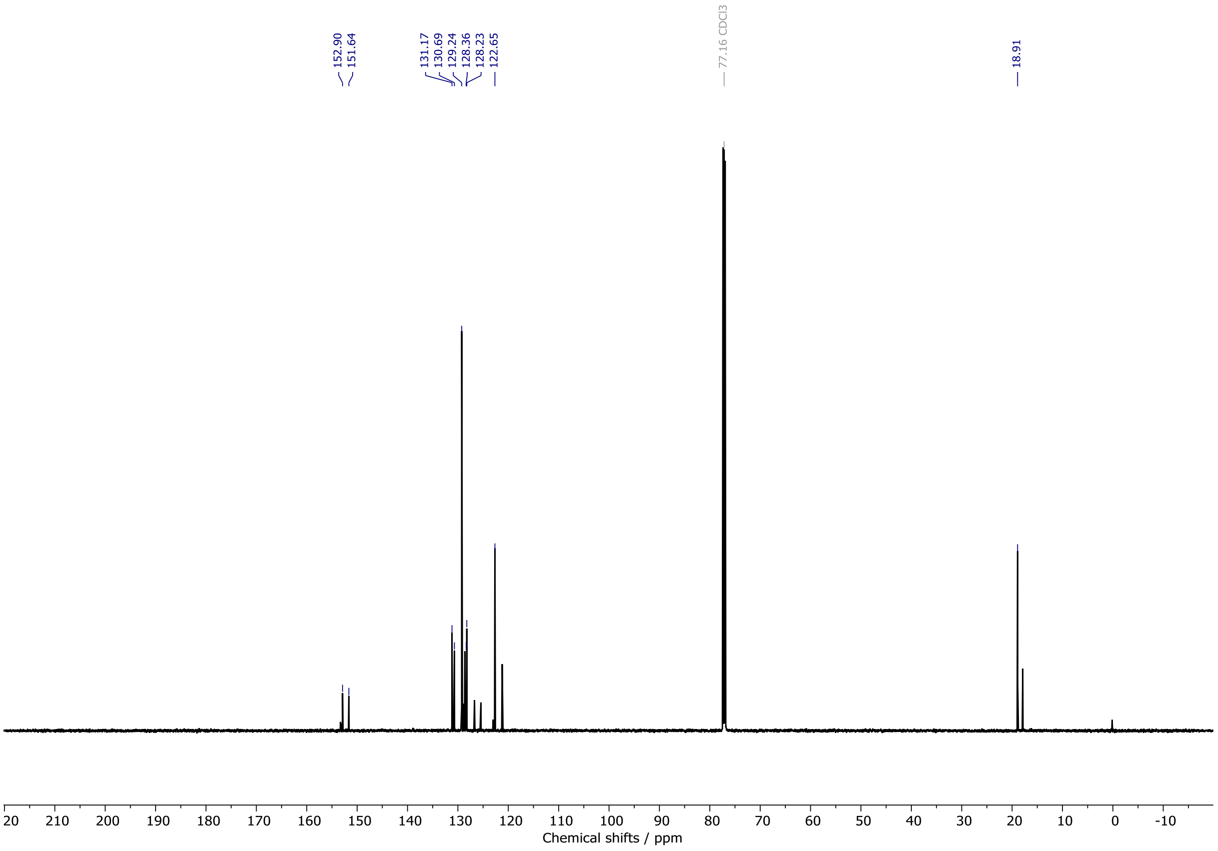

1-(3,5-Dimethylphenyl)-2-phenyldiazene (1l)

^{1}H NMR

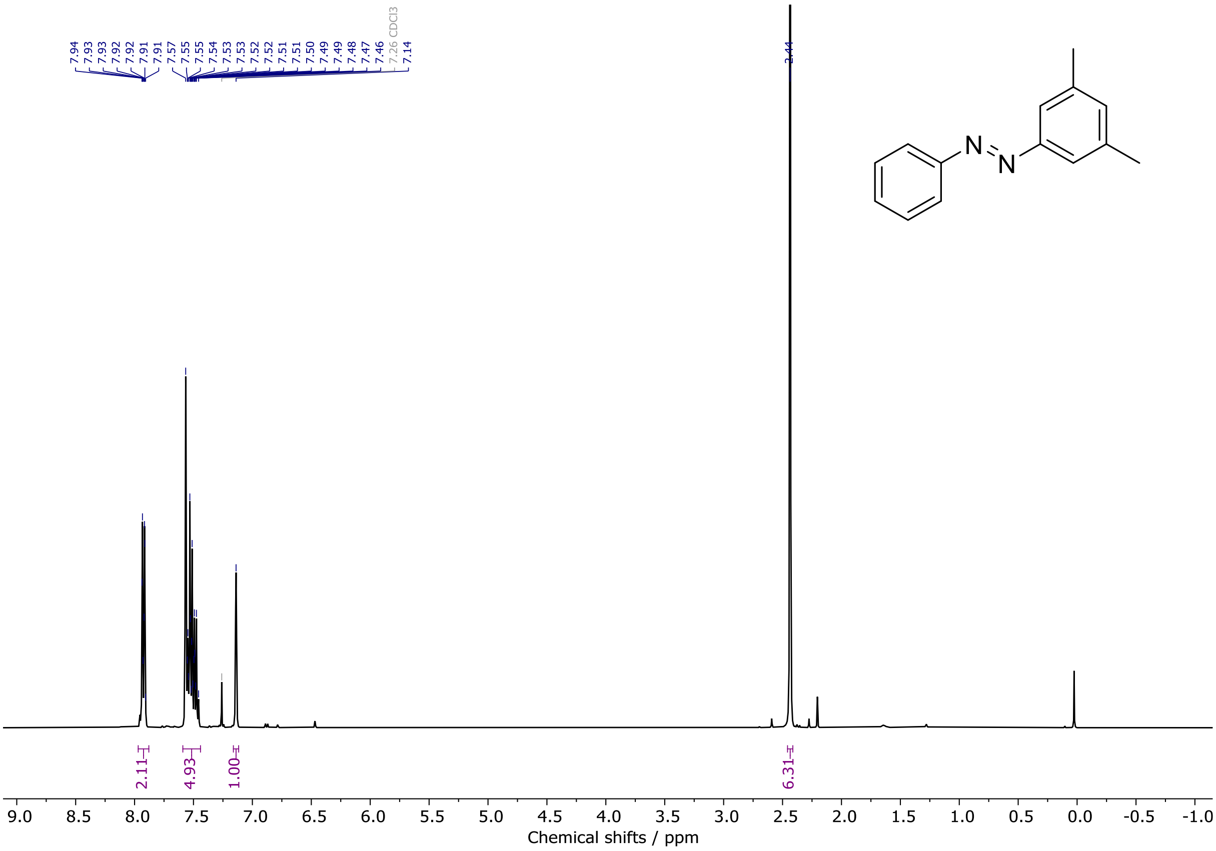

^{13}C NMR

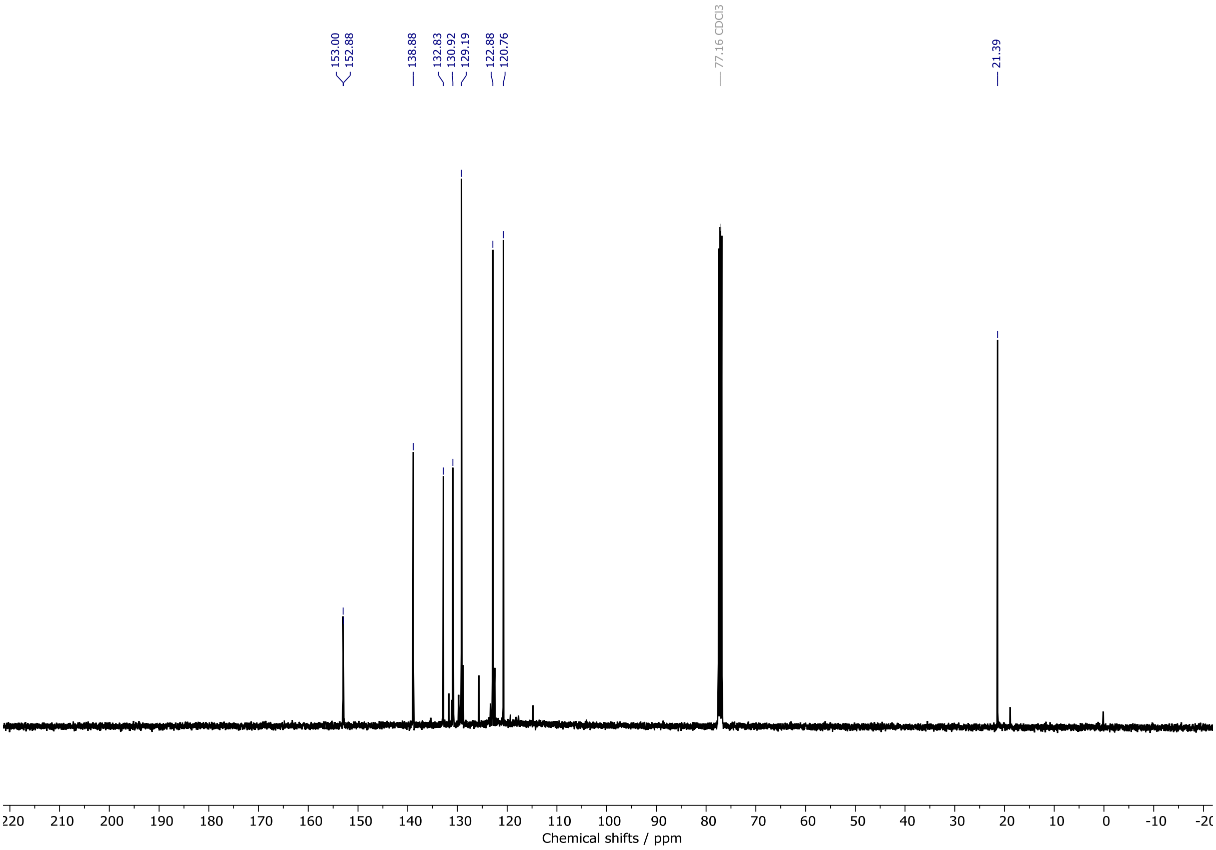

1-Mesityl-2-phenyldiazene (1m)

^{1}H NMR

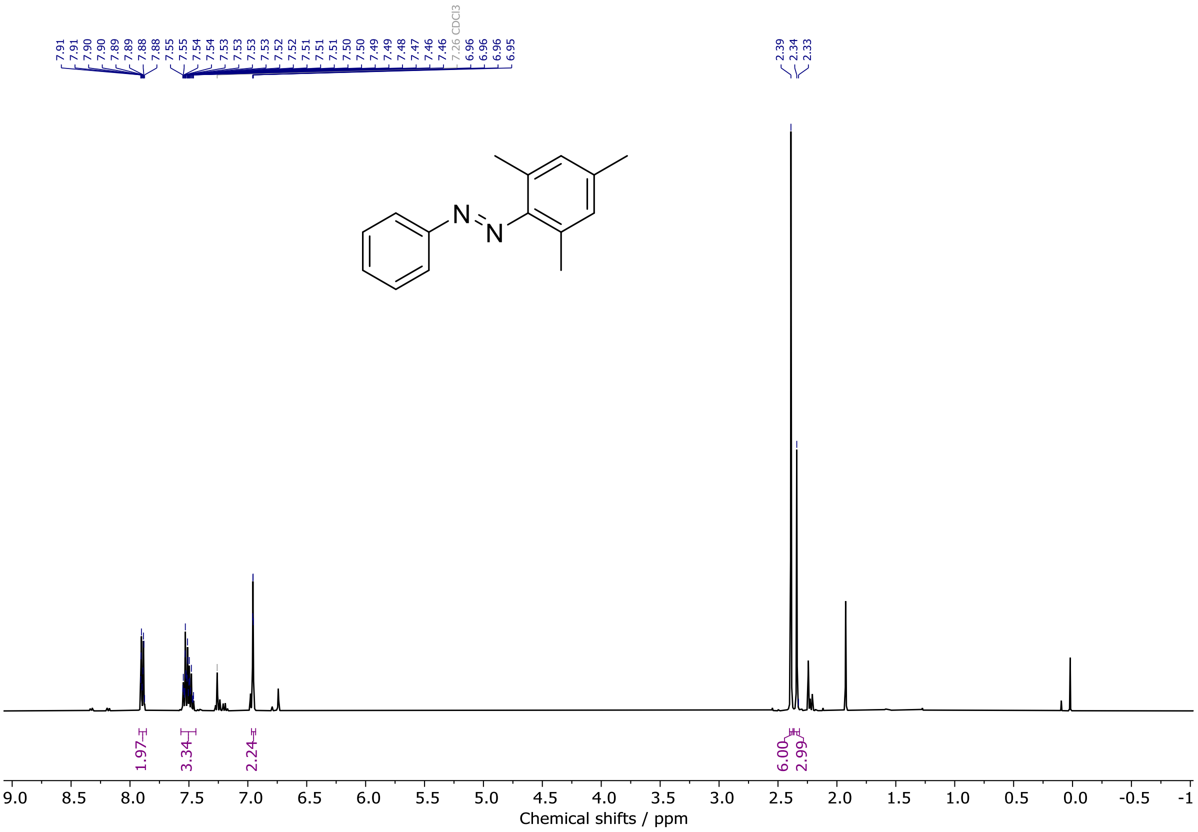

^{13}C NMR

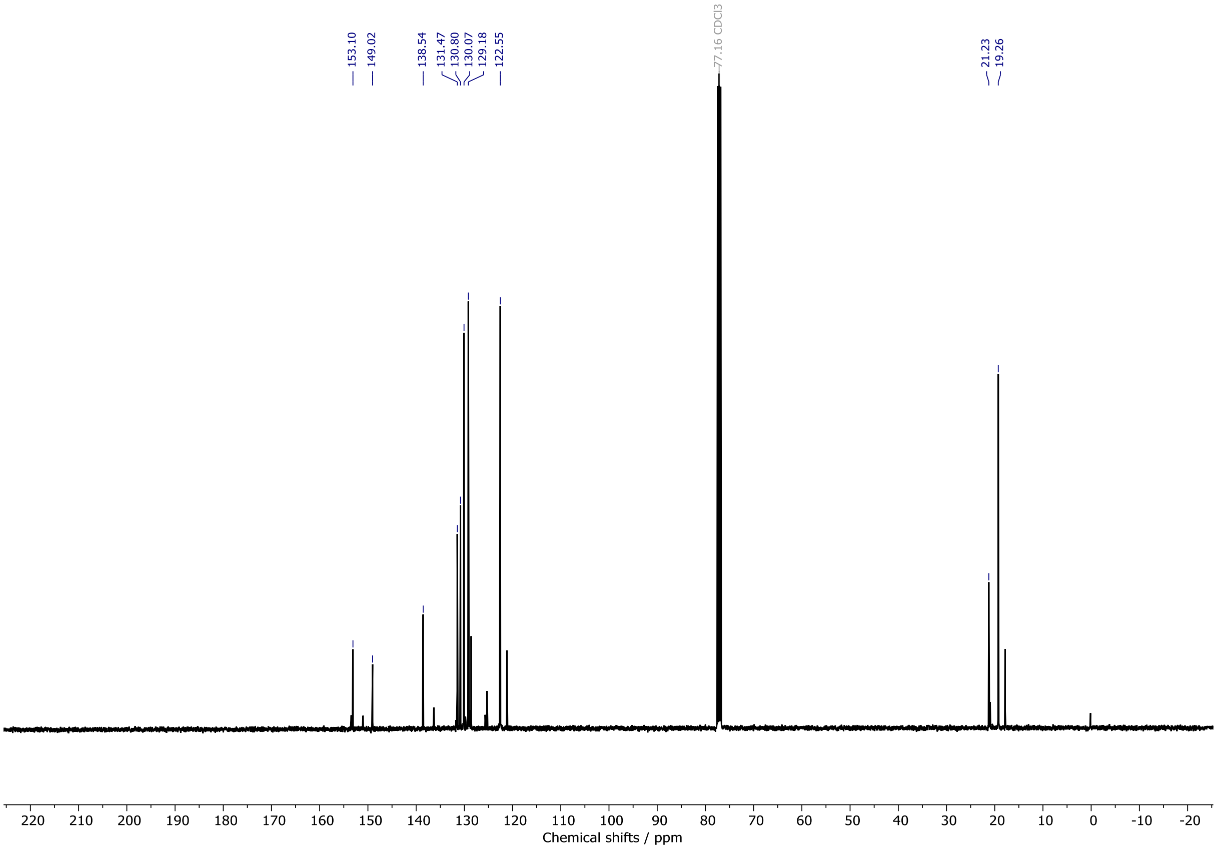

1-(3,5-Di-*tert*-butylphenyl)-2-phenyldiazene (1n)

^{1}H NMR

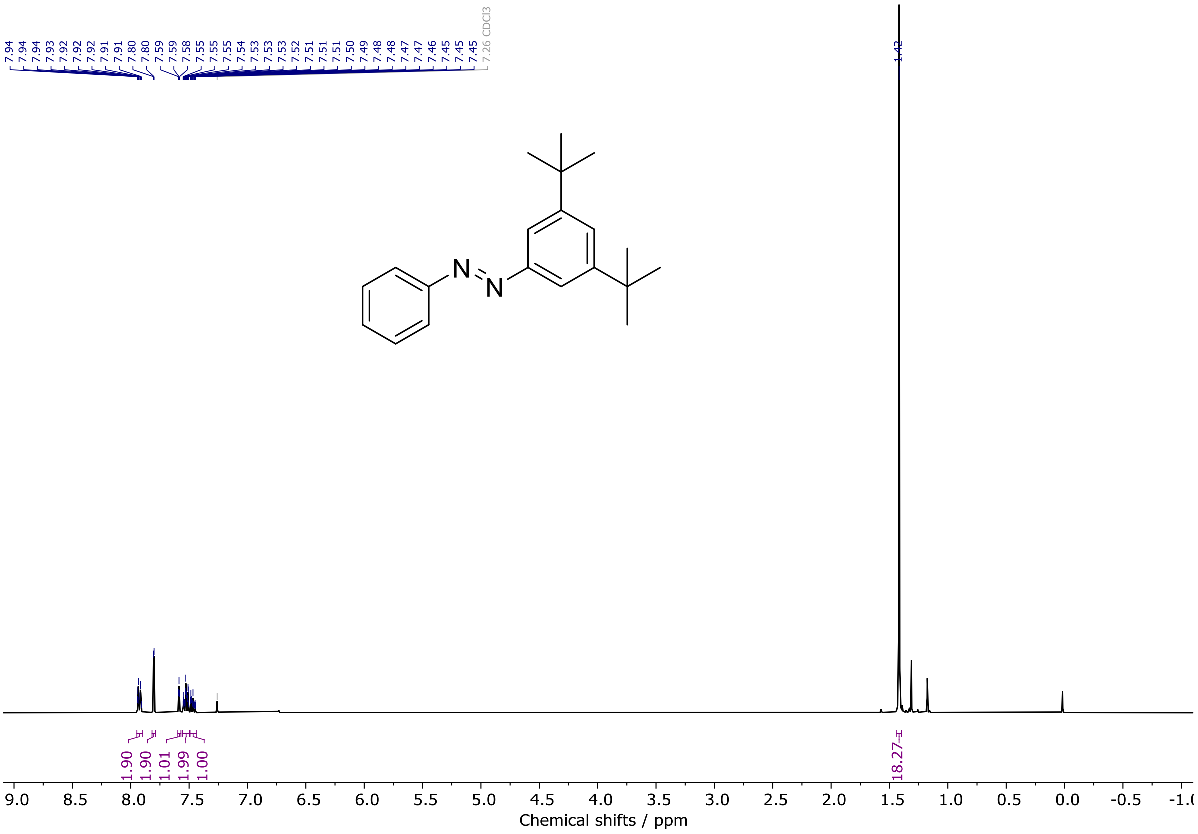

^{13}C NMR

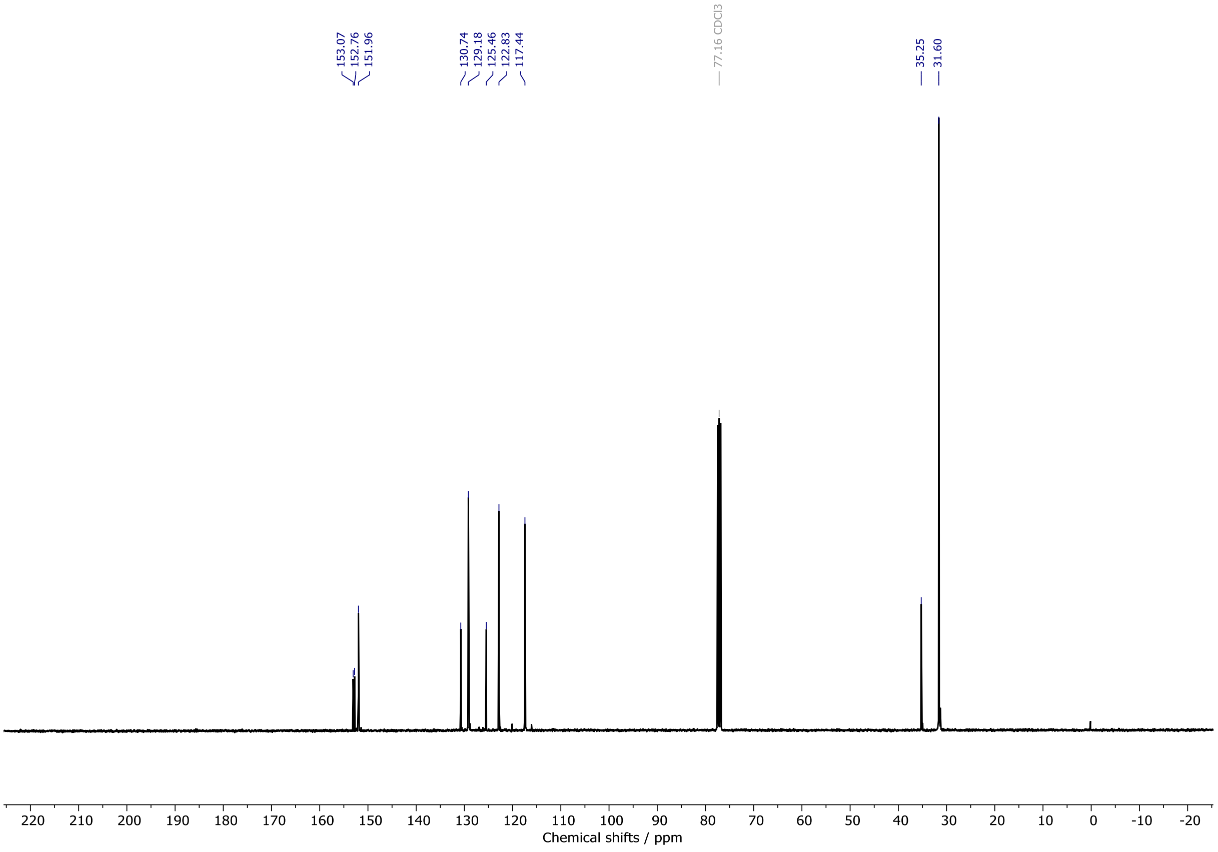

4-(2-Phenyldiazenyl)benzonitrile (1o)

<u>^{1}H NMR</u>

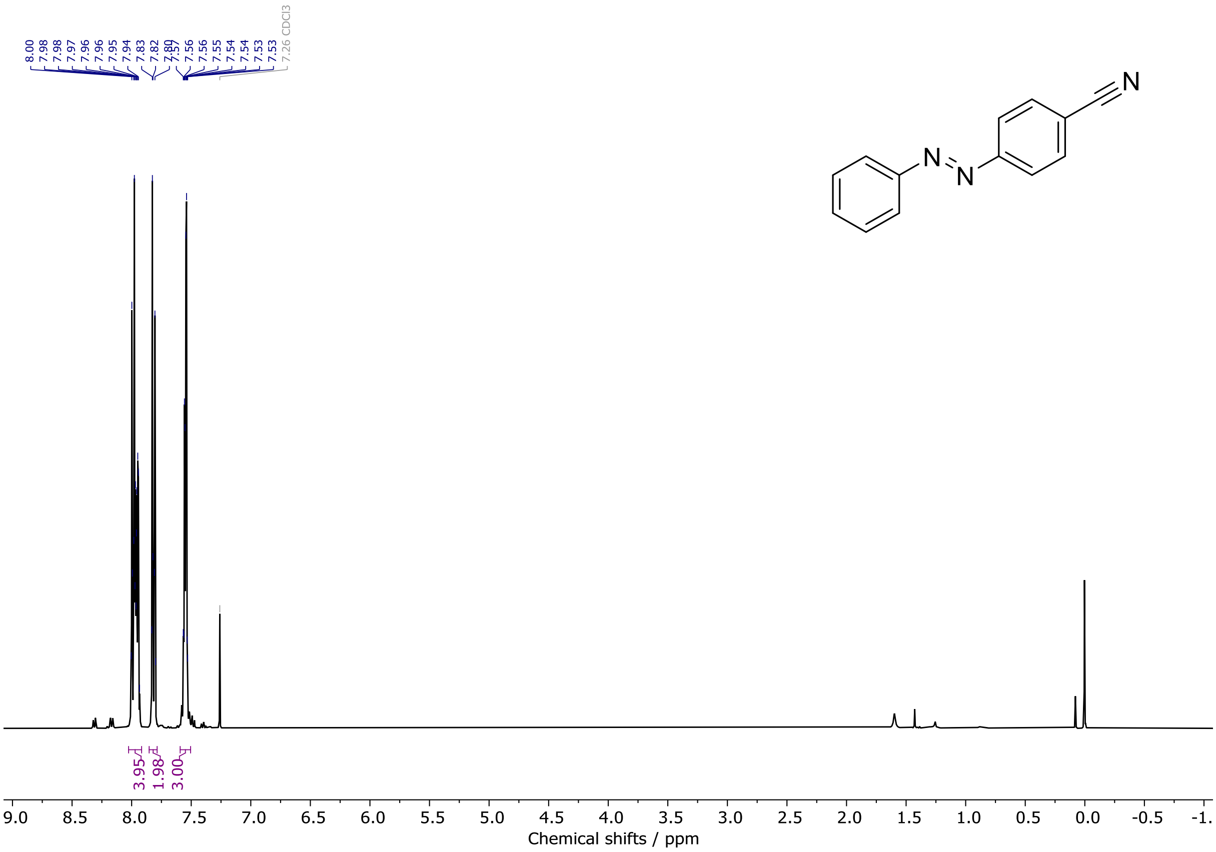

<u>^{13}C NMR</u>

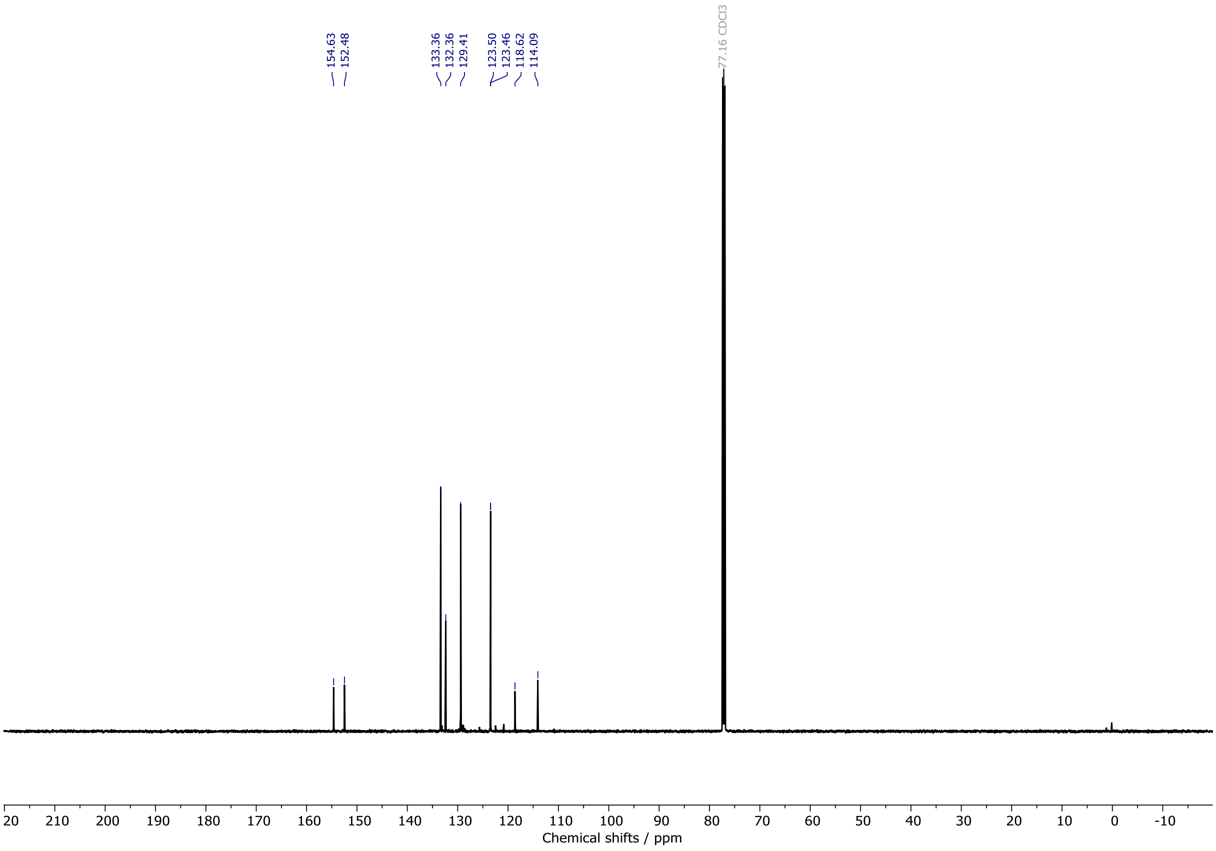

3-(2-Phenyldiazenyl)benzonitrile (1p)

^{1}H NMR

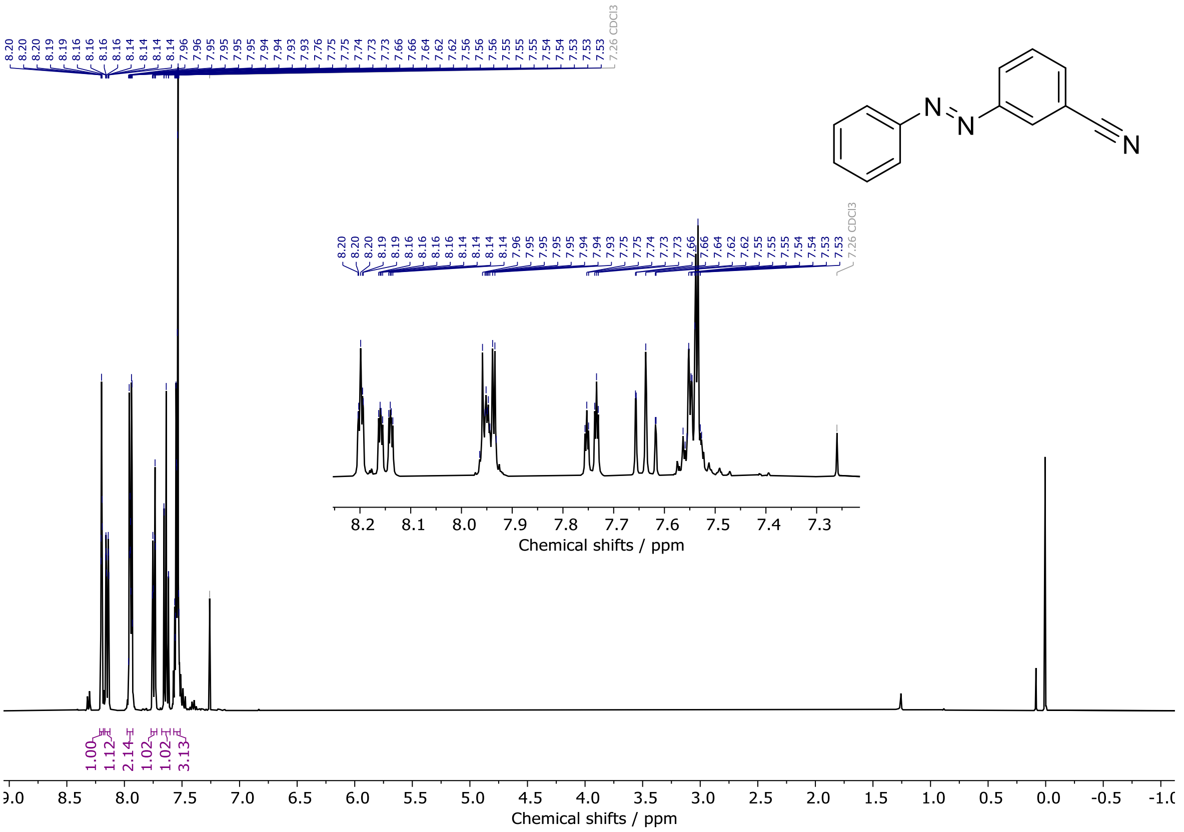

^{13}C NMR

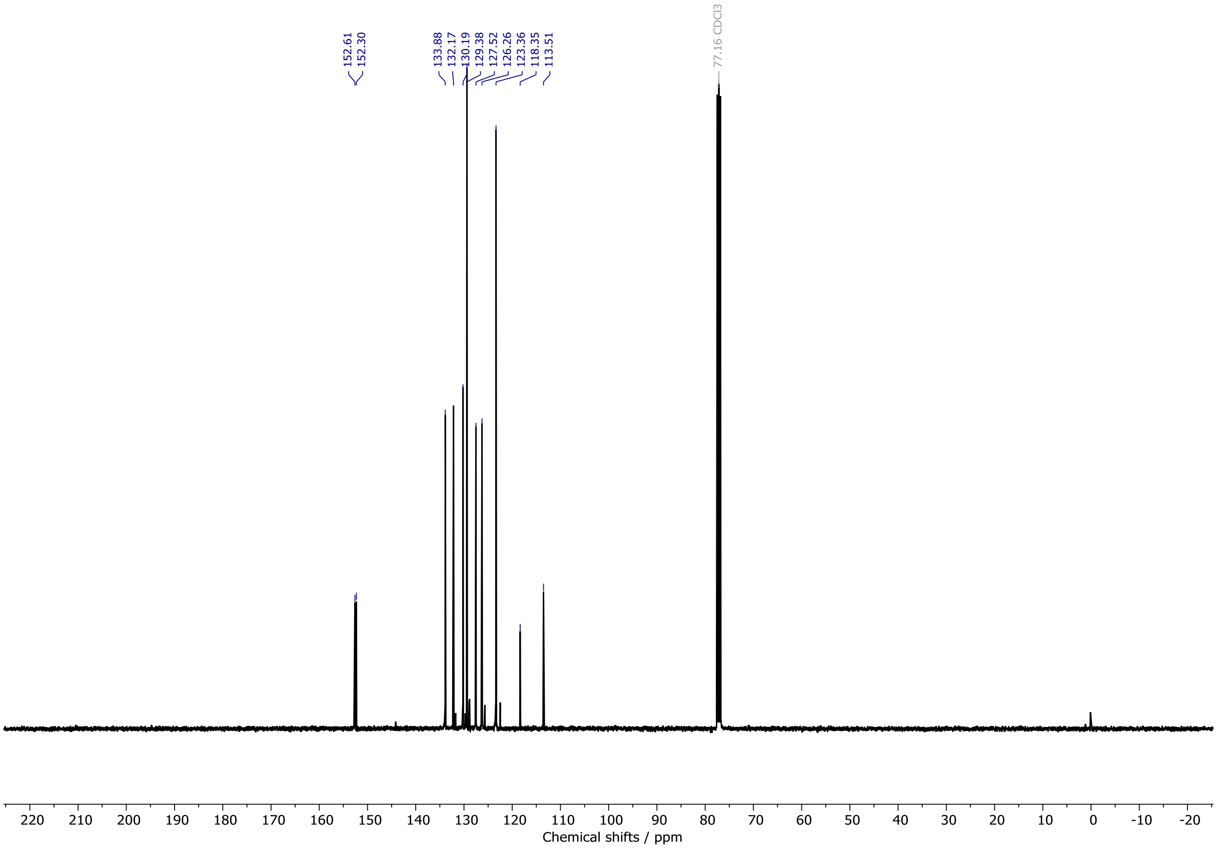

4-Phenylazophenol (1q)

^{1}H NMR

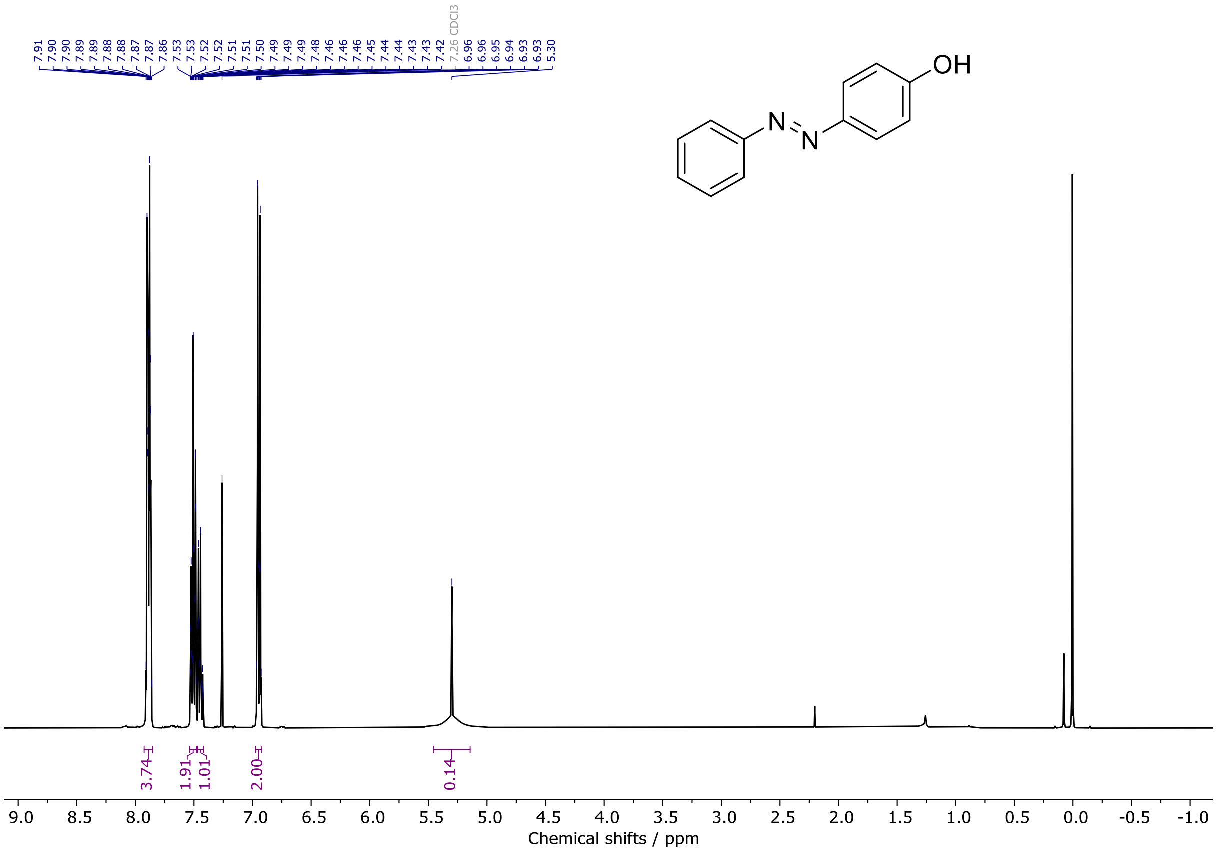

^{13}C NMR

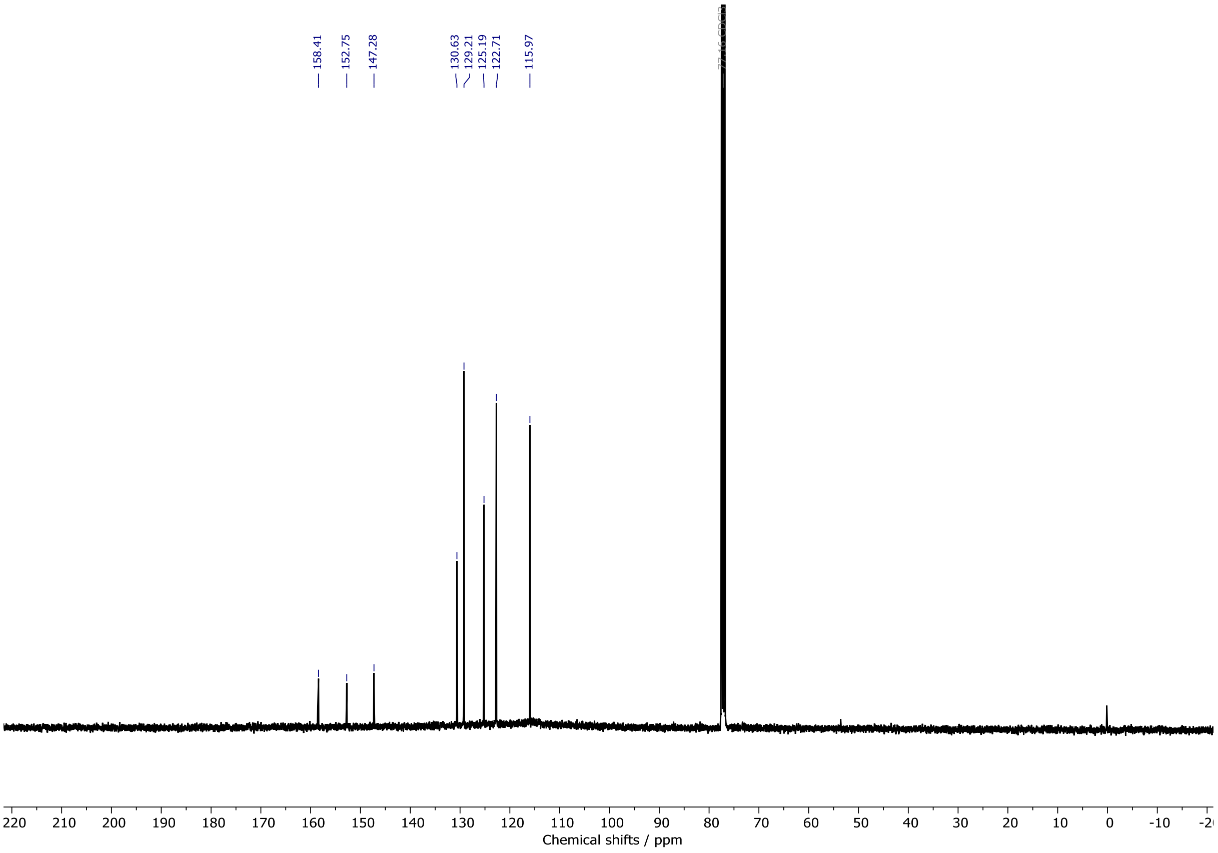

1-Phenyl-2-(4-(trifluoromethyl)phenyl)diazene (1r)

^{1}H NMR

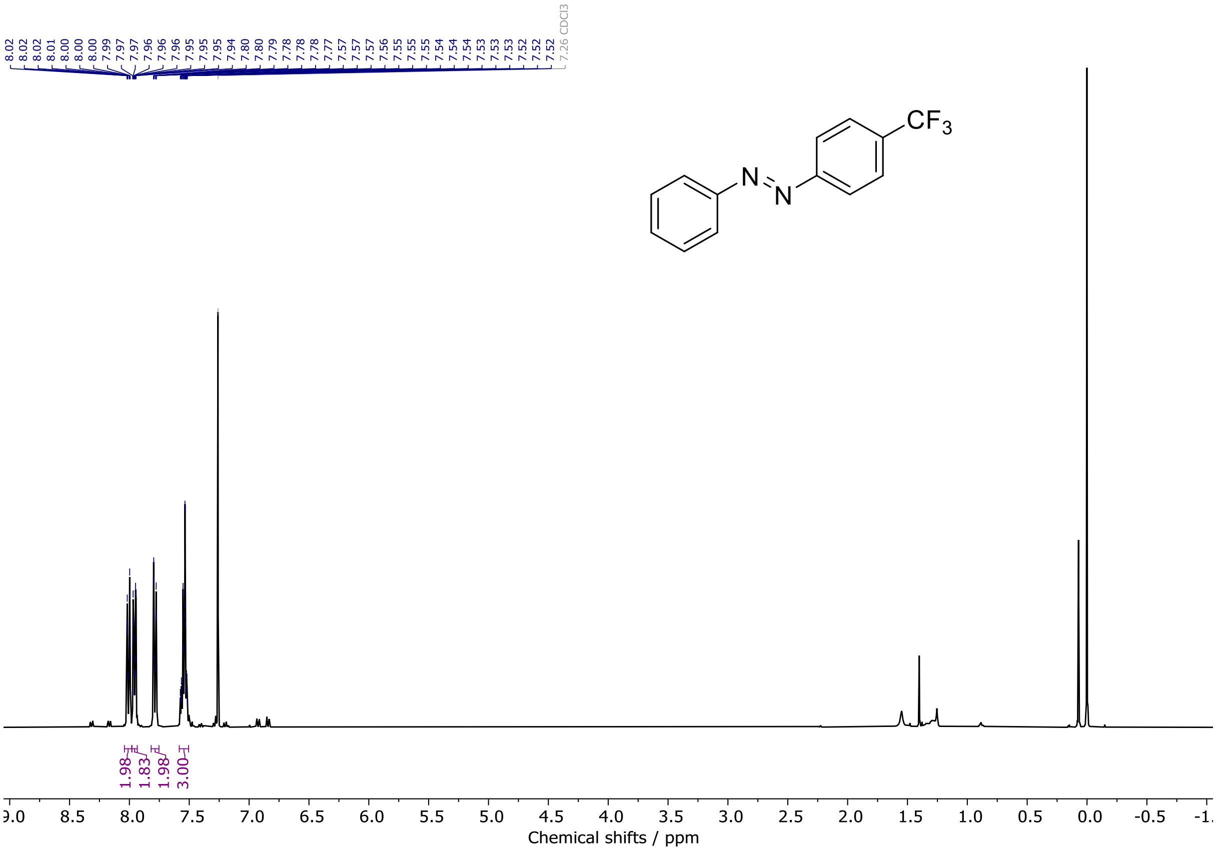

^{13}C NMR

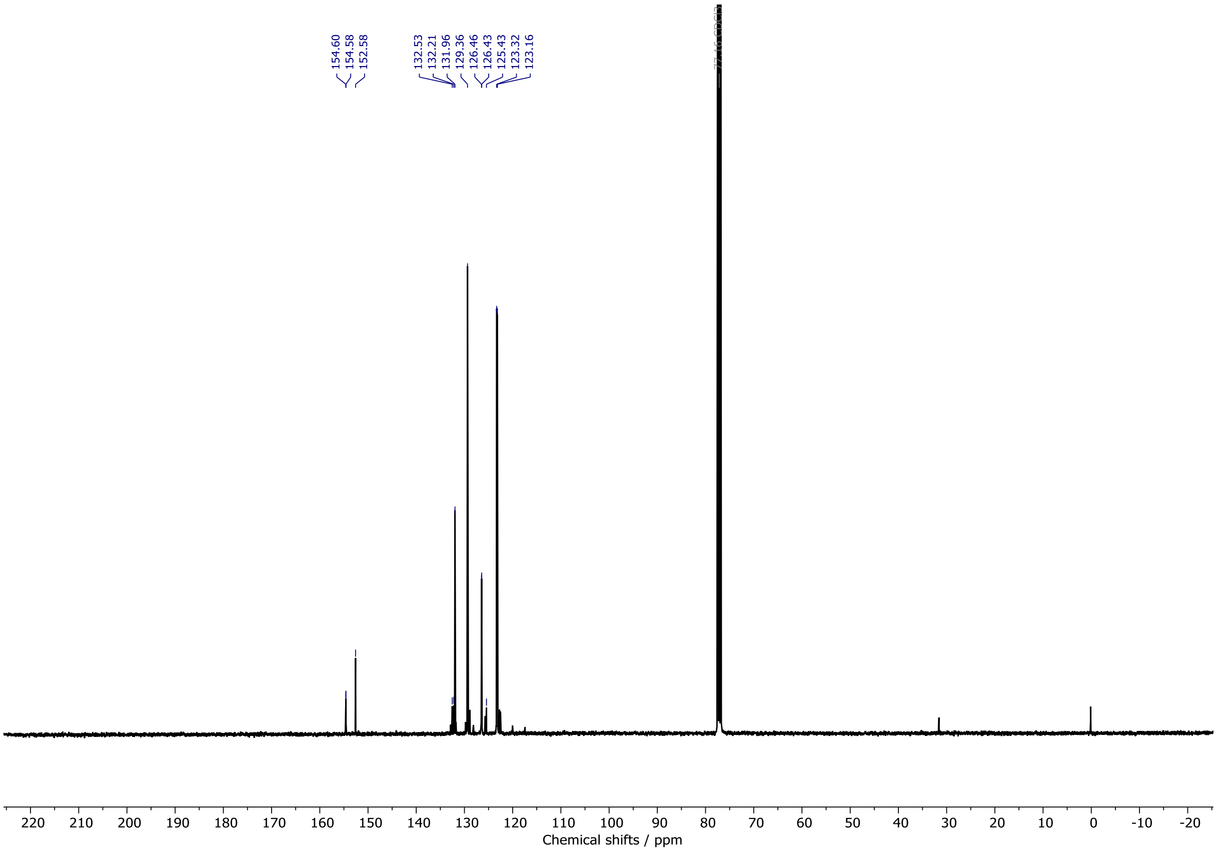

1-(4-Fluorophenyl)-2-phenyldiazene (1s)

^{1}H NMR

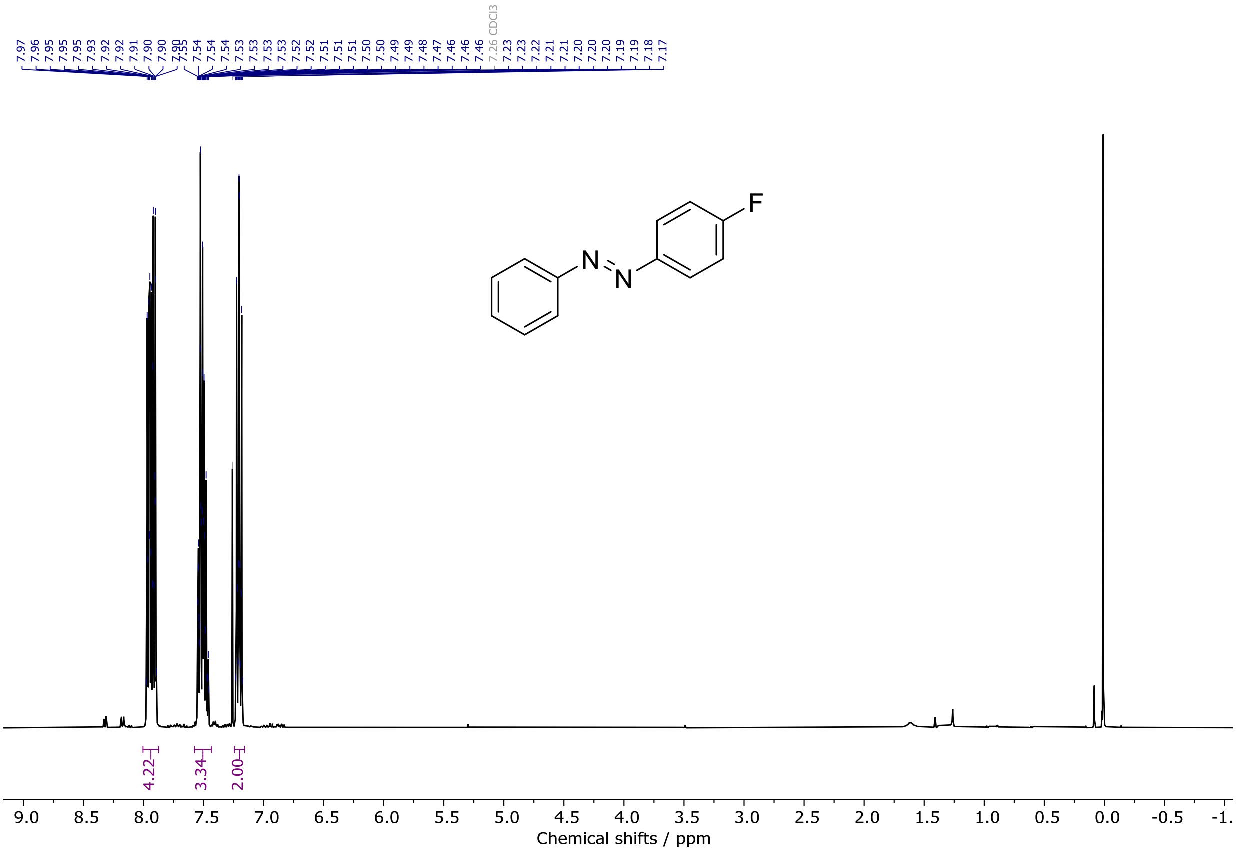

^{13}C NMR

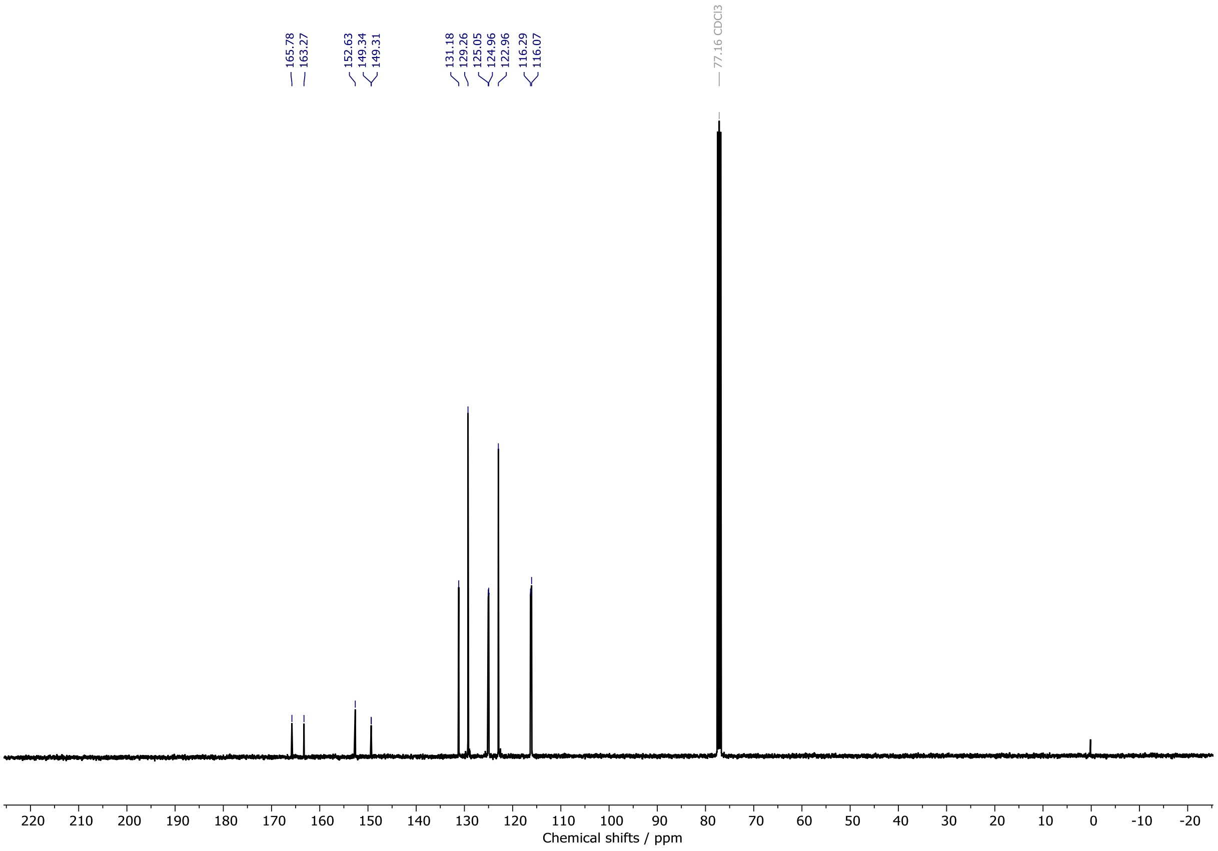

1-[4-[(2-Ethylhexyl)oxy]phenyl]-2-phenyldiazene (1t)

^{1}H NMR

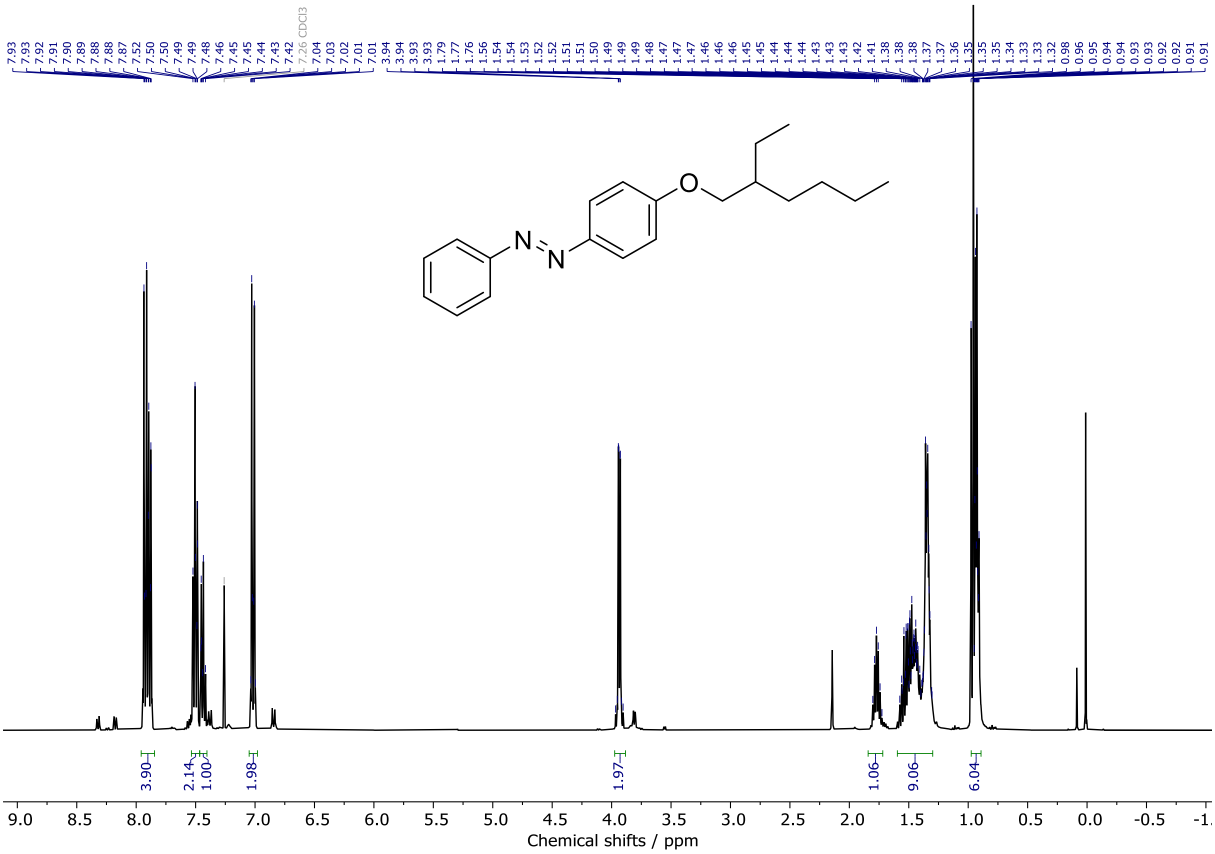

^{13}C NMR

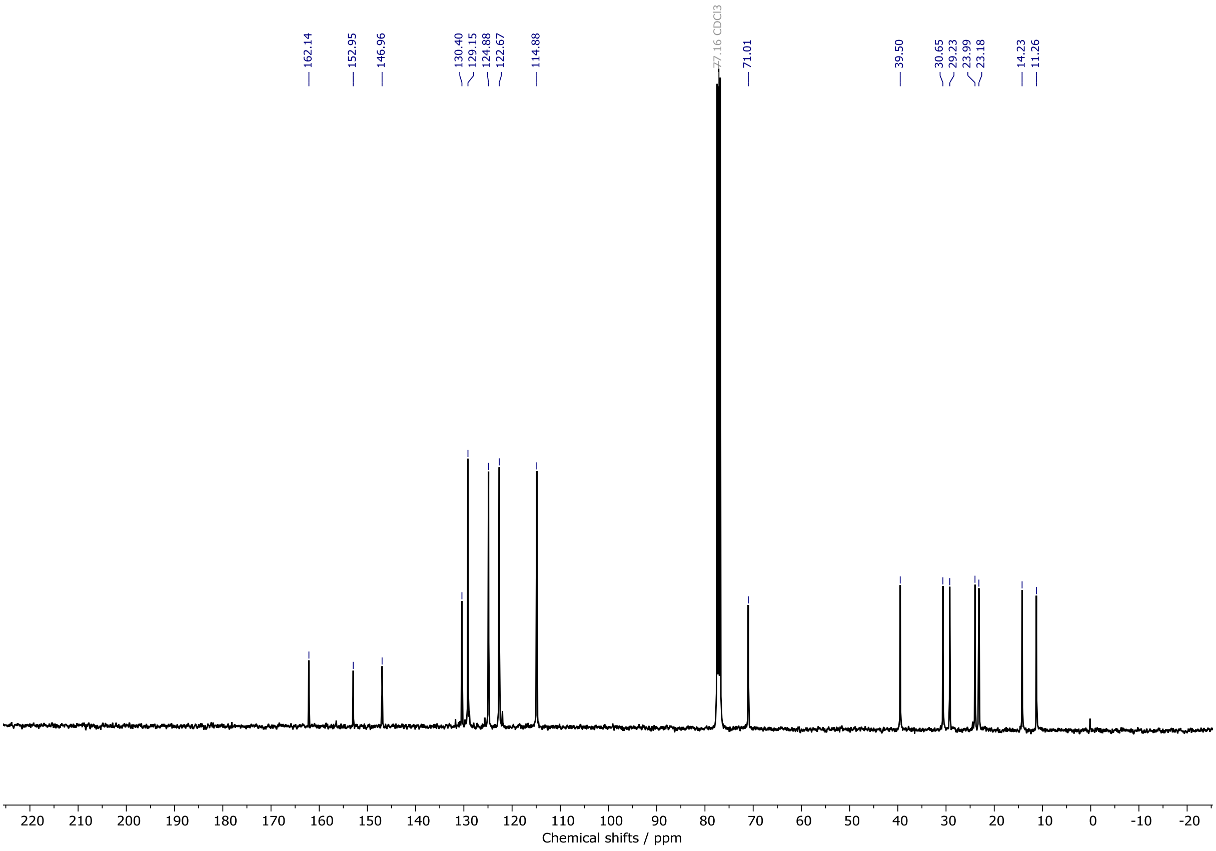

4-(2-Phenyldiazenyl)benzonitrile (1o) – temperature screening

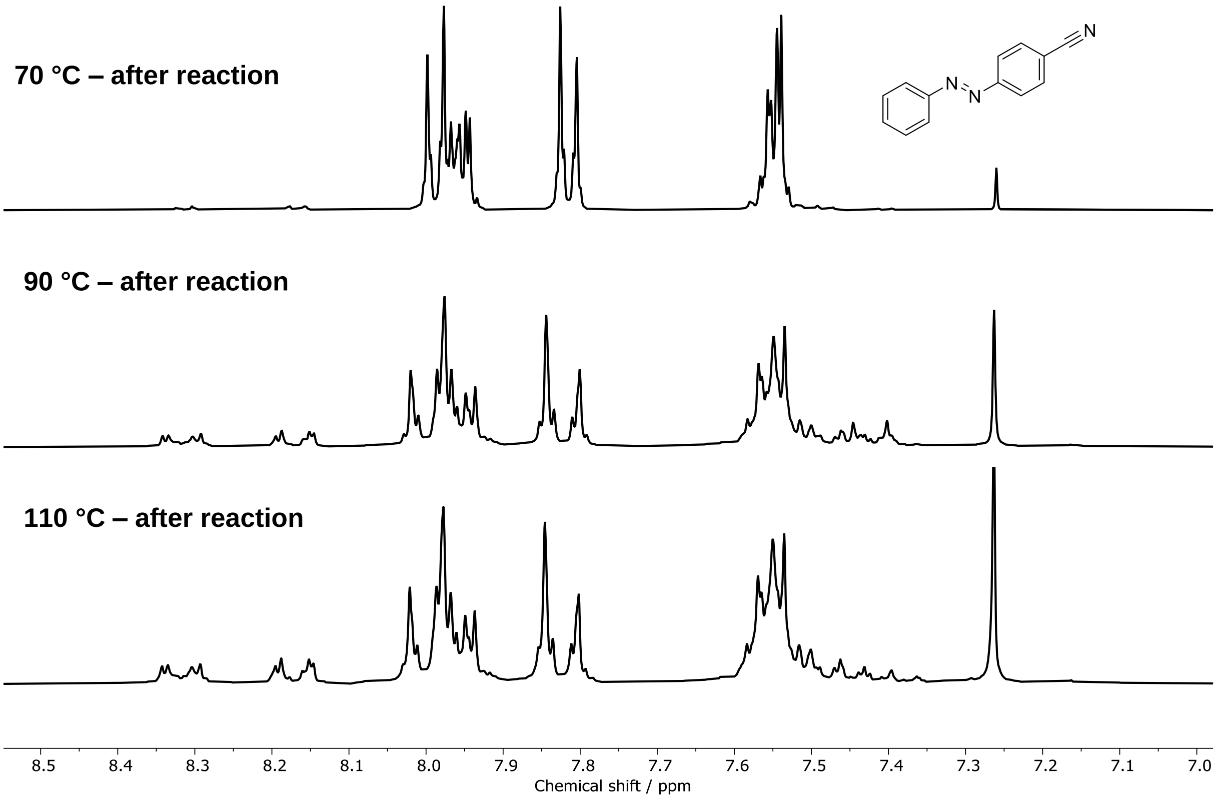

References

1. Kirkus, M.; Knippenberg, S.; Beljonne, D.; Cornil, J.; Janssen, R. A. J.; Meskers, S. C. J. *J. Phys. Chem. A* **2013,** *117* (13), 2782–2789. doi:10.1021/jp400256s
2. Hu, L.; Cao, X.; Shi, L.; Qi, F.; Guo, Z.; Lu, J.; Gu, H. *Org. Lett.* **2011,** *13* (20), 5640–5643. doi:10.1021/ol202362f
3. Denonne, F.; Seiler, P.; Diederich, F. *Helv. Chim. Acta* **2003,** *86* (9), 3096–3117. doi:10.1002/hlca.200390252
4. Lv, H.; Laishram, R. D.; Li, J.; Zhou, Y.; Xu, D.; More, S.; Dai, Y.; Fan, B. *Green Chem.* **2019,** *21* (15), 4055–4061. doi:10.1039/C9GC01235D
5. Zubar, V.; Dewanji, A.; Rueping, M. *Org. Lett.* **2021,** *23* (7), 2742–2747. doi:10.1021/acs.orglett.1c00659
6. Zhao, R.; Tan, C.; Xie, Y.; Gao, C.; Liu, H.; Jiang, Y. *Tetrahedron Lett.* **2011,** *52* (29), 3805–3809. doi:10.1016/j.tetlet.2011.05.054
7. Yi, X.; Jiao, L.; Xi, C. *Org. Biomol. Chem.* **2016,** *14* (41), 9912–9918. doi:10.1039/c6ob01827k
8. Strauss, M. A.; Wegner, H. A. *Angew. Chem. Int. Ed.* **2019,** *58* (51), 18552–18556. doi:10.1002/anie.201910734
9. Tombari, R. J.; Tuck, J. R.; Yardeny, N.; Gingrich, P. W.; Tantillo, D. J.; Olson, D. E. *Org. Biomol. Chem.* **2021,** *19* (35), 7575–7580. doi:10.1039/d1ob01450a
10. Roling, P. V. *J. Org. Chem.* **1975,** *40* (17), 2421–2425. doi:10.1021/jo00905a001
11. Christoforou, D.; Happer, D. A. *Aust. J. Chem.* **1983,** *36* (10), 2083–2094. doi:10.1071/CH9832083
12. Xu, H.; Zeng, X. *Bioorganic Med. Chem. Lett.* **2010,** *20* (14), 4193–4195. doi:10.1016/j.bmcl.2010.05.048
13. Masutani, K.; Morikawa, M.; Kimizuka, N. *Chem. Commun.* **2014,** *50* (99), 15803–15806. doi:10.1039/C4CC07713J
14. Liu, Y.; Liu, B.; Guo, A.; Dong, Z.; Jin, S.; Lu, Y. *Molecules* **2011,** *16* (5), 3563–3568. doi:10.3390/molecules16053563
